# A modal derivatives enhanced Rubin substructuring method for geometrically nonlinear multibody systems

**DOI:** 10.1007/s11044-018-09644-2

**Published:** 2018-10-19

**Authors:** Long Wu, Paolo Tiso, Konstantinos Tatsis, Eleni Chatzi, Fred van Keulen

**Affiliations:** 1 Faculty of Mechanical, Maritime and Materials Engineering, Delft University of Technology, Mekelweg 2, 2628CD Delft, The Netherlands; 2 Institute of Mechanical Systems, ETH Zürich, Leonhardstrasse 21, 8092 Zürich, Switzerland; 3 Institute of Structural Engineering, ETH Zürich, Stefano-Franscini-Platz 5, 8903 Zürich, Switzerland

**Keywords:** Geometric nonlinearity, Floating frame of reference, Modal derivatives, Rubin substructuring, Mean-axis frame

## Abstract

This paper presents a novel model order reduction technique for 3D flexible multibody systems featuring nonlinear elastic behavior. We adopt the mean-axis floating frame approach in combination with an enhanced Rubin substructuring technique for the construction of the reduction basis. The standard Rubin reduction basis is augmented with the modal derivatives of both free-interface vibration modes and attachment modes to consider the bending–stretching coupling effects for each flexible body. The mean-axis frame generally yields relative displacements and rotations of smaller magnitude when compared to the one obtained by the nodal-fixed floating frame. This positively impacts the accuracy of the reduction basis. Also, when equipped with modal derivatives, the Rubin method better considers the geometric nonlinearities than the Craig–Bampton method, as it comprises vibration modes and modal derivatives featuring free motion of the interface. The nonlinear coupling between free-interface modes and attachment modes is also considered. Numerical tests confirm that the proposed method is more accurate than Craig–Bampton’s, a nodal fixed floating frame counterpart originally proposed in Wu and Tiso (Multibody Syst. Dyn. 36(4): 405–425, [2016]), and produces significant speed-ups. However, the offline cost is increased because the mean-axis formulation produces operators with decreased sparsity patterns.

## Introduction

The simulation of flexible multibody systems (FMBS) often relies on finite element (FE) discretization of flexible components, which are then embedded into a floating frame of reference (FFR) formulation [2, 3]. The FFR represents the position of each body as a superposition of two components: (i) the motion of the reference frame which follows the overall rigid body motion of the flexible body; (ii) the relative motion of the flexible body with respect to the reference frame. The floating frame approach is usually preferred to the description of the multibody motion with respect to the inertial frame (see, for instance, [4]) as it naturally distinguishes the elastic deformation from the rigid body motion. The resulting models often comprise a large number of degrees of freedom (DoFs), which render time integration schemes extremely costly. A relevant example of unaffordable computational burden could be found in the simulation of large-scale offshore wind turbines. To assess their fatigue life, thousands of load cases need to be simulated, resulting in disproportionally large computation times. At present, this can be achieved only by relying on extremely simplified beam models that reduce the computational cost to a bearable level. Such models do not inherit the complexity of the actual three-dimensional model of the blade, and, as a result, the complex dynamic behavior may not be appropriately represented. For this reason, many model order reduction (MOR) strategies for three-dimensional FMBS have been proposed in the past. These techniques are based on classic modal truncation [5, 6] or singular value decomposition (SVD) based MOR techniques as in [7–9]. In [10], a global modal parametrization based MOR method is proposed, where the motion of the FMBS is described in terms of configuration dependent modes. Using this reduction method, the nonlinear holonomic constraints are naturally satisfied without the adoption of Lagrange Multipliers. However, in most of the MOR techniques, the elastic behavior is assumed to be linear. As discussed in [5], the linear MOR with FFR formulation is only suitable for structures featuring large rigid body motions but small relative displacements with respect to the reference frame, as well as slow rotational speeds. For FMBS featuring high rigid body rotation rates, the centrifugal force is of great significance, and therefore, the centrifugal stiffening effect and foreshortening effect have to be considered.

For many FMBS applications involving finite but moderate relative rotations with respect to the reference frame, neglecting geometrical nonlinearities may lead to incorrect and even diverging solutions [11, 12]. In [13], the geometrical nonlinearities are introduced in the equations of motion. As a result, the internal force vector and tangent matrix need to be recomputed for every iteration within each time step, therefore significantly impacting the computational cost. It is then a must to extend the linear MOR methods to the geometrically nonlinear regime for three-dimensional FMBS.

When one substructure of the FMBS features geometrically nonlinear behavior, dominant low-frequency modes are not sufficient for adequately representing the relative motion with respect to the reference frame. Typically, large slender structures exhibit coupling between bending and axial displacements when excited in the nonlinear regime. The corresponding bending–stretching coupling could be in principle provided by adding membrane-dominant (usually high-frequency) modes to the bending-dominant (typically low-frequency) modes based reduction basis. For flat structures, where each vibration mode exhibits purely bending or membrane displacement, such membrane modes can be easily identified and added to the reduced-order basis (ROB). The inclusion of these so-called *ad hoc* modes has been applied in the FFR formulation in [14, 15]. However, for more complex geometries, the extraction of such modes is (i) challenging, as it is not straightforward to identify membrane-dominated modes, and (ii) expensive, as several modes need to be extracted.

In previous work [1], the linear Craig–Bampton (CB) substructuring basis [16] was enriched with modal derivatives (MDs) [17, 18] corresponding to low-frequency fixed-interface modes. The augmented ROB was capable of capturing both the rigid body motions and the nonlinear relative displacement of the FMBS effectively. The nonlinear MOR technique was applied for nodal-fixed frame reference [19], which is the most straightforward implementation of the FFR formulation. In this case, the reference frame is attached to specified nodes of the moving body. However, for complex structures, e.g., discretized with shell and solid elements, it is difficult to determine the optimal node whereon the reference frame should be attached. This arbitrary definition of the nodal-fixed frame results in significantly different relative displacements and rotations with respect to the reference frame [19], and ultimately degrades the accuracy if the relative displacement and rotations are too large.

The use of mean-axis frame [20], which alleviates the need for the reference frame to be attached to a specified node of the structure, aims at minimizing the relative kinetic energy with respect to the reference frame. As a result, the largest relative displacement and rotation observed from a mean-axis frame will be smaller than the largest one observed when standing at the origin of the nodal-fixed frame, as underlined in [19]. This is especially relevant when one assumes geometrical nonlinearities based on the von Kármán kinematic assumption, which is suitable for small strains and moderate rotations [21] with respect to the reference frame. Since the MDs are obtained from a truncated Taylor expansion of the nonlinear static equilibrium around the reference position [22, 23] and are not updated during the time integration, the accuracy of using MDs will be determined by how far the structure departs from the equilibrium position. Therefore, the use of MDs further supports the argument of using the mean-axis formulation.

In this paper, the standard Rubin substructuring technique [24] is enhanced with MDs and then implemented on the mean-axis frame formulation for the construction of reduced-order models (ROMs) for the FMBS featuring moderate relative displacements and rotations with respect to the reference frame. Each body is reduced by forming the ROB with attachment modes, free-interface modes, and corresponding MDs. The Rubin method fits the mean-axis formulation more naturally than the CB method when applied to the geometrically nonlinear problem, for two reasons. First, the Rubin method is based on a truncated set of free-interface vibration modes, which naturally describe the elastic deformation of the component with respect to the reference frame (i.e., free-interface deformation with respect to the reference frame as in mean-axis frame formulation). Second, the nonlinear behavior occurring at the interface is better represented by MDs of both free-interface modes and attachment modes (related to the Rubin method) than by MDs of fixed interface modes coming from the CB method. In [25], the inclusion of only the MDs relative to rigid body modes (i.e., vibration modes of zero frequency) in the ROB significantly increases the accuracy. In our approach, the MDs relative to rigid body modes are avoided since the rigid body motion has already been described by the reference frame motion. Therefore, a ROB of very limited size can be achieved.

This paper is organized as follows. Section 2 describes the FFR description featuring geometric nonlinearities. The nodal-fixed and mean-axis frame are applied to the FFR formulation in Sect. 3. The assembled EoMs of all FMBS, as well as the holonomic joint constraints, are presented in Sect. 4. The nonlinear MOR method based on the enhanced Rubin method is proposed in Sect. 5. Section 6 shows numerical examples to assess the accuracy of the present formulation, especially emphasizing the improvements with respect to [1]. Finally, conclusions are given in Sect. 7.

## Equations of motion in floating frame of reference

**Fig. 1:**
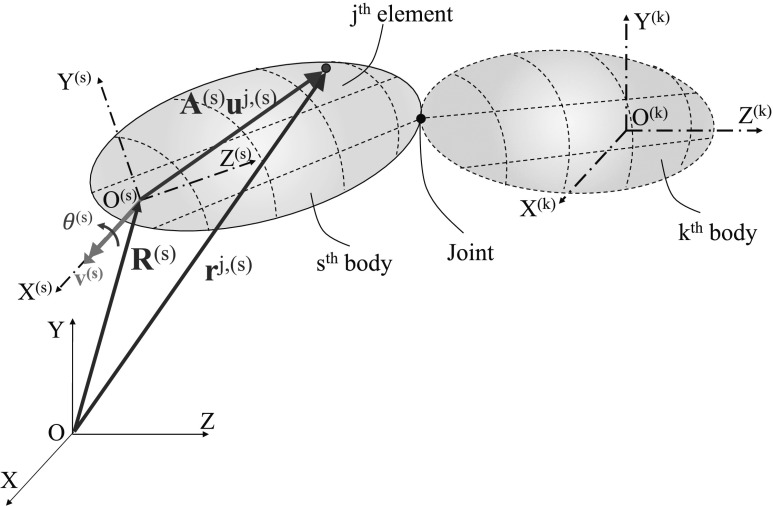
Generalized coordinates expression in floating frame of reference. Two bodies are coupled through a joint. The absolute position of an arbitrary point (red) in the $j$th element of the $s$th body is shown (Color figure online)

In the FFR formulation, we describe the absolute motion of an arbitrary point $P^{j,(s)}$ on the $j$th finite element of the $s$th body as the superposition of the motion of the reference frame $O^{(s)}X^{(s)}Y ^{(s)}Z^{(s)}$ and the position of the point with respect to the reference frame, as shown in Fig. 1. The position vector $\mathbf{r}^{j,(s)}\in \mathbb{R}^{3}$ of the point $P^{j,(s)}$ is defined as

1 $$ \mathbf{r}^{j,(s)}= \mathbf{R}^{(s)}+ \mathbf{A}^{(s)}\mathbf{u}^{j,(s)} =\mathbf{R}^{(s)}+ \mathbf{A}^{(s)}\mathbf{N}^{j,(s)} \bigl(\mathbf{q}_{0}^{j,(s)}+ \mathbf{q}_{f}^{j,(s)} \bigr) , $$

where $\mathbf{R}^{(s)}\in \mathbb{R}^{3}$ represents the position of origin of the reference frame $O^{(s)}X^{(s)}Y^{(s)}Z^{(s)}$ with respect to global frame $OXYZ$, $\mathbf{u}^{j,(s)} \in \mathbb{R} ^{3}$ is the relative nodal position of $P^{j,(s)}$ with respect to the reference frame, and $\mathbf{A}^{(s)} \in \mathbb{R}^{3\times 3}$ is the transformation matrix from the reference frame $O^{(s)}X^{(s)}Y ^{(s)}Z^{(s)}$ to the global frame $OXYZ$. The matrix of shape functions in the reference frame is indicated by $\mathbf{N}^{j,(s)}\in \mathbb{R}^{3\times n_{e}}$, where $n_{e}$ is the number of DoFs per element, $\mathbf{q}_{0}^{j,(s)} \in \mathbb{R}^{n_{e}}$ is the vector of nodal coordinates in the undeformed state and $\mathbf{q}_{f}^{j,(s)} \in \mathbb{R}^{n_{e}}$ is the vector of relative DoFs of the $j$th element. For the remainder of this paper, we drop the superscript $\star ^{(s)}$ for the sake of clarify, unless it is necessary to distinguish between different bodies in the FMBS.

The rotation matrix $\mathbf{A}$ is defined as

2 $$ \mathbf{A}= \begin{bmatrix} 1-2\theta _{2}^{2}-2\theta _{3}^{2} & 2(\theta _{1}\theta _{2}-\theta _{0}\theta _{3}) & 2(\theta _{1}\theta _{3}+\theta _{0}\theta _{2})\\ 2(\theta _{1}\theta _{2}+\theta _{0}\theta _{3}) & 1-2\theta _{1}^{2}-2 \theta _{3}^{2} & 2(\theta _{2}\theta _{3}-\theta _{0}\theta _{1})\\ 2(\theta _{1}\theta _{3}-\theta _{0}\theta _{2}) & 2(\theta _{2}\theta _{3}+\theta _{0}\theta _{1}) & 1-2\theta _{1}^{2}-2\theta _{2}^{2} \end{bmatrix} , $$

where the four Euler parameters $\boldsymbol{\theta }=\operatorname{col}( \theta _{0}, \theta _{1}, \theta _{2}, \theta _{3})$ are used:

$$\begin{aligned} \theta _{0} =&\cos \frac{\theta }{2}, \qquad \theta _{1}=v_{1} \sin \frac{\theta }{2}, \qquad \theta _{2}=v_{2}\sin \frac{\theta }{2}, \\ \theta_{3} =&v_{3}\sin \frac{\theta }{2}, \quad \text{with } \theta _{0}^{2}+\theta _{1}^{2}+\theta _{2}^{2}+\theta _{3}^{3}=1. \end{aligned}$$

Here, the notation $\operatorname{col}(...)$ indicates the column stacking of vectors or scalar quantities. The unit vector along the rotation axis is given by $\mathbf{v}=\operatorname{col}(v_{1},v_{2},v_{3})$ and $\theta $ is the rotation angle. The axis of rotation along $\mathbf{v}$ and the rotation angle $\theta $ are defined for each flexible body separately.

The absolute velocity $\dot{\mathbf{r}}^{j}$ is given by

3 $$\begin{aligned} \dot{\mathbf{r}}^{j} &=\dot{ \mathbf{R}}+\dot{\mathbf{A}}\mathbf{N} ^{j}\bigl(\mathbf{q}_{0}^{j}+ \mathbf{q}_{f}^{j}\bigr)+\mathbf{A}\mathbf{N}^{j} \dot{\mathbf{q}}_{f}^{j} \\ &= \begin{bmatrix} \mathbf{I} & \boldsymbol{\mathcal{B}}^{j}\{\boldsymbol{\theta }, \mathbf{q}_{f}^{j}\} & \mathbf{A}\mathbf{N}^{j} \end{bmatrix} \begin{bmatrix} \dot{\mathbf{R}}\\ \dot{\boldsymbol{\theta }}\\ \dot{\mathbf{q}}_{f} ^{j} \end{bmatrix}, \end{aligned}$$

where the argument of functional dependency is enclosed in braces. The second term in (3) is rewritten as

4 $$ \dot{\mathbf{A}}\mathbf{N}^{j}\bigl(\mathbf{q}_{0}^{j}+ \mathbf{q}_{f}^{j}\bigr)= \boldsymbol{\mathcal{B}}^{j} \bigl\{ \boldsymbol{\theta }, \mathbf{q}_{f} ^{j}\bigr\} \dot{ \boldsymbol{\theta }}, $$

in order to isolate the velocity terms $\dot{\boldsymbol{\theta }}$. The matrix $\boldsymbol{\mathcal{B}}^{j}$ is thus a function of $\boldsymbol{\theta }$, $\mathbf{q}_{f}^{j}$ and $\mathbf{q}_{0}^{j}$. The dependency of $\boldsymbol{\mathcal{B}}^{j}$ on $\mathbf{q}_{0} ^{j}$ will not be explicitly expressed since $\mathbf{q}_{0}^{j}$ is constant for each FE model.

The kinetic energy $\mathcal{T}^{j}$ for the $j$th element can be evaluated by

5 $$ \mathcal{T}^{j}=\frac{1}{2} \int _{V^{j}} \rho ^{j}\bigl(\dot{\mathbf{r}}^{j} \bigr)^{ \text{T}}\dot{\mathbf{r}}^{j}\,\mathrm{d}V^{j} = \frac{1}{2}\bigl(\dot{\mathbf{q}}^{j}\bigr)^{ \text{T}} \mathbf{M}^{j}\dot{\mathbf{q}}^{j}, $$

where $\mathbf{q}^{j} = \operatorname{col}( \mathbf{R} , \boldsymbol{\theta } , \mathbf{q}^{j}_{f}) $ and $\rho ^{j}$ is the density of the element material. The mass matrix $\mathbf{M}^{j}$ is configuration-dependent (its formulation is given in detail in Appendix A). The kinetic energy of the $s$th body can be determined by summing up the kinetic energy $\mathcal{T}^{j}$ of all its elements. The mass matrix $\mathbf{M}$ of the $s$th body is obtained by standard FE assembly. The vector $\mathbf{q}=\operatorname{col}( \mathbf{R} , \boldsymbol{\theta } , \mathbf{q}_{f})$ indicates the generalized coordinates of a single flexible body, where $\mathbf{q} _{f}\in \mathbb{R}^{n}$ refers to the total relative DoFs in the reference frame and $n$ is the number of relative DoFs.

The equations of motion (EoMs) for each flexible body can be derived from Lagrange’s equations as

6 $$ \frac{\mathrm{d}}{\mathrm{d}t} \biggl( \frac{\partial \mathcal{T}}{\partial \dot{\mathbf{q}}} \biggr) ^{\text{T}}- \biggl( \frac{\partial \mathcal{T}}{\partial \mathbf{q}} \biggr) ^{\text{T}}+ \biggl( \frac{\partial \mathcal{U}}{\partial \mathbf{q}} \biggr) ^{\text{T}} = \mathbf{g}, $$

where $\mathcal{T}$ and $\mathcal{U}$ are the kinetic energy and strain energy, respectively; $\mathbf{g}$ is the vector of externally applied generalized loads. At this stage, the flexible bodies (if more than one) have not been assembled and the prescribed motions of the flexible bodies have not been imposed yet.

The EoMs (6) can be rewritten in matrix form as

7 $$ \mathbf{M}\{\mathbf{q}\}\ddot{\mathbf{q}}-\mathbf{Q}\{\mathbf{q}, \dot{\mathbf{q}}\}+\mathbf{f}\{\mathbf{q}\} =\mathbf{g}, $$

where $\mathbf{Q}$ is the quadratic velocity vector, which includes the effect of apparent forces (such as centrifugal force and Coriolis force), and $\mathbf{f}$ is the nonlinear elastic force vector. The quadratic velocity vector $\mathbf{Q}$ results from the inertia coupling between the motion $\operatorname{col}(\mathbf{R}, \boldsymbol{\theta })$ of the reference frame and the relative motion $\mathbf{q}_{f}$. The derivation of $\mathbf{Q}$ is given in Appendix A.

In this work, we adopt the von Kármán kinematic assumption for geometric nonlinearities, which is suitable for small strains and moderate rotations [21]. The elastic force $\mathbf{f}$ can be directly derived from the differentiation of the strain energy and may be written as a third-order polynomial function of the relative DoFs $\mathbf{q}_{f}$.

Equation (7) can be conveniently written in a partitioned form that highlights the coupling between $\operatorname{col}(\mathbf{R}, \boldsymbol{\theta })$ and $\mathbf{q}_{f}$ as

8 $$ \begin{bmatrix} \mathbf{M}_{RR} & \mathbf{M}_{R\theta } & \mathbf{M}_{Rf}\\ & \mathbf{M}_{\theta \theta } & \mathbf{M}_{\theta f}\\ \text{sym} & & \mathbf{M}_{ff} \end{bmatrix} \begin{bmatrix} \ddot{\mathbf{R}}\\ \ddot{\boldsymbol{\theta }}\\ \ddot{\mathbf{q}}_{f} \end{bmatrix} - \begin{bmatrix} \mathbf{Q}_{R}\\ \mathbf{Q}_{\theta }\\ \mathbf{Q}_{f} \end{bmatrix} + \begin{bmatrix} \mathbf{0}\\ \mathbf{0}\\ \mathbf{f}_{f} \end{bmatrix} = \begin{bmatrix} \mathbf{g}_{R}\\ \mathbf{g}_{\theta }\\ \mathbf{g}_{f} \end{bmatrix} , $$

where the explicit dependency on $\mathbf{q}$ is dropped for clarity. The subscripts $\star _{R}$, $\star _{\theta }$ and $\star _{f}$ indicate the partitions corresponding to $\mathbf{R}$, $\boldsymbol{\theta }$ and $\mathbf{q}_{f}$, respectively.

**Fig. 2:**
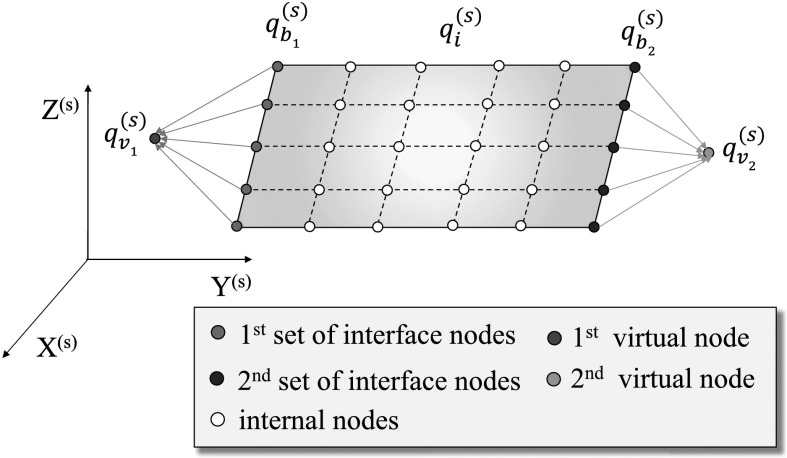
Illustration of the “rigidizing” of the interface sets of the $s$th body. The flexible body contains two sets of interface DoFs. The relative DoFs $\mathbf{q}^{(s)}_{b_{1}}$, $\mathbf{q}^{(s)}_{b_{2}}$ of the two interface sets are described by the relative DoFs $\mathbf{q}^{(s)}_{v_{1}}$, $\mathbf{q}^{(s)}_{v_{2}}$ of their corresponding virtual nodes w.r.t. the reference frame, respectively (Color figure online)

In this work, we rigidize the interface by rigidly linking each interface set with a reference virtual node, and expressing all relative interface DoFs $\mathbf{q}_{b_{p}}$ at the $p$th interface set through 6 DoFs $\mathbf{q}_{v_{p}} \in \mathbb{R}^{6}$ (3 translational DoFs and 3 rotational DoFs) of the virtual node, as illustrated in Fig. 2. The 6 DoFs $\mathbf{q}_{v_{p}}$ represent the relative translations and rotations of the $p$th virtual node with respect to the reference frame. The rigid body constraints are commonly applied at the interface, when the flexible bodies are connected through rigid joints.

To be specific, we split the relative DoFs $\mathbf{q}_{f}\in \mathbb{R}^{n}$ in the $s$th body into the sets of relative interface DoFs $\mathbf{q}_{b} \in \mathbb{R}^{n_{b}}$ and relative internal DoFs $\mathbf{q}_{i} \in \mathbb{R}^{n_{i}} $ as

9 $$ \mathbf{q}_{f}=\operatorname{col}(\mathbf{q}_{b} , \mathbf{q}_{i}) = \operatorname{col}(\mathbf{q}_{b_{1}} , \dots , \mathbf{q}_{b_{w}} , \mathbf{q}_{i}), $$

where the interface DoFs $\mathbf{q}_{b}$ have been further divided into different interface sets from $\mathbf{q}_{b_{1}} \in \mathbb{R}^{n _{b_{1}}}$ to $\mathbf{q}_{b_{w}} \in \mathbb{R}^{n_{b_{w}}}$, and $w$ is the number of interface sets. It holds that $n_{b_{1}}+\cdots +n _{b_{w}}=n_{b}$. The transformation from DoFs $\mathbf{q}_{b}$ of all interface nodes to the DoFs $\mathbf{q}_{v}$ of all virtual nodes can be written as

10 $$ \mathbf{q}_{b} = \begin{bmatrix} \mathbf{q}_{b_{1}}\\ \vdots\\ \mathbf{q}_{b_{w}} \end{bmatrix} = \underbrace{ \begin{bmatrix} \mathbf{L}_{v_{1}} & &\\ & \ddots &\\ & & \mathbf{L}_{v_{w}} \end{bmatrix} } _{ \mathbf{L}_{v} } \underbrace{ \begin{bmatrix} \mathbf{q}_{v_{1}}\\ \vdots\\ \mathbf{q}_{v_{w}} \end{bmatrix} } _{\mathbf{q}_{v}}, $$

where $\mathbf{L}_{v} \in \mathbb{R}^{n_{b} \times n_{v}}$ is the transformation matrix of the entire interface DoFs, and $n_{v}$ is the number of DoFs for all virtual nodes. Matrix $\mathbf{L}_{v}$ is formed according to the position of each interface node. The detailed expression of $\mathbf{L}_{v}$ is given in Appendix B.

It should be noticed that the FE discretized components, without imposed constraints, allow relative rigid body motion of the flexible bodies with respect to the body reference frame. In the FFR formulation, however, the rigid body motion has already been described by the translation and rotation of the reference frame. To define a unique displacement field, we need to eliminate redundant DoFs, by imposing a set of reference constraints. This is discussed in the next section.

## Floating frame definition

We now briefly summarize the nodal-fixed definition [19] and the mean-axis definition [20] of the FFR, together with the embedding technique utilized to impose the constraints introduced by the mean-axis frame definition.

### Nodal-fixed frame

**Fig. 3:**
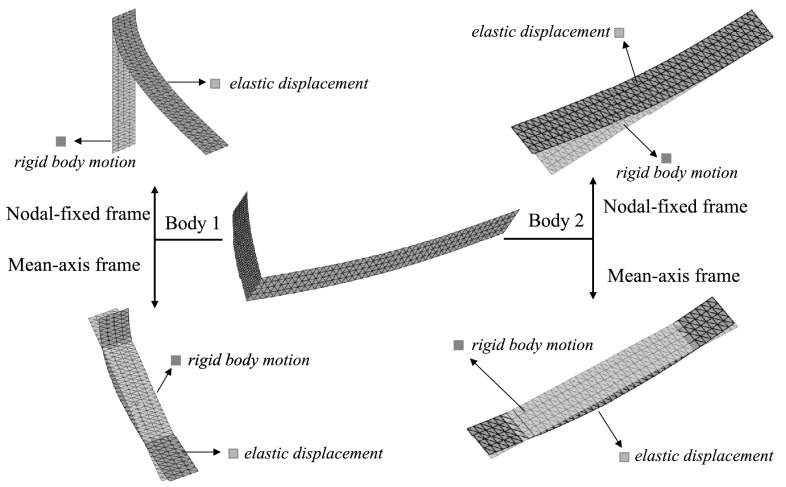
The kinematic description of a crank–shaft system (middle) for both nodal-fixed frame (top) and mean-axis frame (bottom) (Color figure online)

The nodal-fixed frame is commonly applied since its definition is straightforward. In this work, the origin of the reference frame is attached to a specified node of the moving body, i.e., no relative translations and rotations of the attached node with respect to the reference frame are allowed. Clearly, the choice of this attached node is not unique. Here, we choose the virtual node of the $k$th interface set. Mathematically, this is simply done by fixing the 6 relative DoFs $\mathbf{q}_{v_{k}}$ of the corresponding virtual node with respect to the reference frame as

11 $$ \mathbf{q}_{v_{k}}=\mathbf{0}, $$

which corresponds to 6 scalar constraints. For illustration, we show the kinematic description of a crank–shaft system for the nodal-fixed floating frame in Fig. 3. The gray mesh denotes the rigid body motion of each body, defined by the position and orientation of the reference frame. When “standing” at the origin of the reference frame, one observes the relative displacements and rotations of the body as the flexible body is clamped at the origin of the frame.

### Mean-axis frame

If an approximated kinematic model for only moderate relative rotations formulation is adopted, as the case of the von Kármán model in the present work, one should try to keep the relative rotations with respect to the reference frame as small as possible. Since the magnitude of the relative displacements and rotations with respect to the nodal-fixed frame largely depends on the choice of the attachment node, the mean-axis floating frame is a more clever choice. Unlike the nodal-fixed frame, the mean-axis frame imposes constraint condition as a function of relative DoFs at each body impartially. The basic idea is to locate the reference frame in such a way that the relative kinetic energy is minimum with respect to an observer stationed on the reference frame. The relative kinetic energy of the $j$th element in the $s$th flexible body is defined as [26]

12 $$ \mathcal{T}_{r}=\sum_{j} \mathcal{T}^{j}_{r}=\sum_{j} \frac{1}{2} \int _{V^{j}}\rho ^{j}\bigl(\dot{\mathbf{u}}_{f}^{j} \bigr)^{\text{T}} \dot{\mathbf{u}}_{f}^{j} \,\mathrm{d}V^{j}. $$

According to (3), the relative velocity $\dot{\mathbf{u}}_{f}^{j}$ of an arbitrary point in the $j$th element is rewritten by stating

13 $$ \dot{\mathbf{u}}_{f}^{j}= \mathbf{N}^{j}\dot{\mathbf{q}}_{f}^{j}= \mathbf{A}^{\text{T}} \bigl[ \dot{\mathbf{r}}^{j}-\dot{\mathbf{R}}- \boldsymbol{\mathcal{B}}^{j}\dot{\boldsymbol{\theta }} \bigr] . $$

Therefore, the relative kinetic energy $\mathcal{T}_{r}$ can be expressed as

14 $$ \mathcal{T}_{r} =\sum_{j} \frac{1}{2} \int _{V^{j}}\rho ^{j} \bigl[ \dot{\mathbf{r}}^{j}- \dot{\mathbf{R}}-\boldsymbol{\mathcal{B}}^{j} \dot{\boldsymbol{\theta }} \bigr] ^{\text{T}} \bigl[ \dot{\mathbf{r}}^{j}-\dot{\mathbf{R}}- \boldsymbol{\mathcal{B}}^{j} \dot{\boldsymbol{\theta }} \bigr] \,\mathrm{d}V^{j}. $$

If $\dot{\mathbf{R}}$ and $\dot{\boldsymbol{\theta }}$ are to satisfy the mean-axis condition, the kinetic energy $\mathcal{T}_{r}$ should be minimum. As discussed, for instance, in [26], we first rewrite the time derivatives of the Euler parameters as a function of the angular velocity vector $\boldsymbol{\omega }$, i.e.,

15 $$ \boldsymbol{\omega }=2\mathbf{E}\dot{\boldsymbol{\theta }}, $$

with

$$ \mathbf{E}= \begin{bmatrix} -\theta _{1} & \theta _{0} & \theta _{3} & -\theta _{2}\\ -\theta _{2} & -\theta _{3} & \theta _{0} & \theta _{1}\\ -\theta _{3} & \theta _{2} & -\theta _{1} & \theta _{0} \end{bmatrix} , \qquad\boldsymbol{\omega} = \begin{bmatrix} \omega _{1}\\ \omega _{2}\\ \omega _{3} \end{bmatrix} = \begin{bmatrix} 2(\theta _{3}\dot{\theta }_{2}-\theta _{2}\dot{\theta }_{3}-\theta _{1} \dot{\theta }_{0}+\theta _{0}\dot{\theta }_{1})\\ 2(\theta _{1}\dot{\theta }_{3}+\theta _{0}\dot{\theta }_{2}-\theta _{3} \dot{\theta }_{1}-\theta _{2}\dot{\theta }_{0})\\ 2(\theta _{2}\dot{\theta }_{1}-\theta _{3}\dot{\theta }_{0}+\theta _{0} \dot{\theta }_{3}-\theta _{1}\dot{\theta }_{2}) \end{bmatrix} . $$

Then, the minimum for $\mathcal{T}_{r}$ can be found by posing

16 $$ \frac{\partial \mathcal{T}_{r}}{\partial \dot{\mathbf{R}}}=\mathbf{0} \quad \text{and} \quad \frac{\partial \mathcal{T}_{r}}{\partial \boldsymbol{\omega}}=\mathbf{0}. $$

Equation (16) yields 6 constraint equations to satisfy the mean-axis condition. In [26], the mean-axis constraint equations are further simplified and finally linearized as a function of $\dot{\mathbf{q}}_{f}$, which is the time derivative of the relative DoFs with respect to reference frame. The approximated mean-axis condition is expressed as

17 $$ \mathbf{S} \dot{\mathbf{q}}_{f} =\mathbf{0}, $$

where $\mathbf{S}\in \mathbb{R}^{6 \times n}$ is a matrix of constant parameters, usually referred to as inertia integrals. The detailed derivation and linearization from (16) to (17) are given in Appendix C.

In order to express the mean-axis condition in terms of $\mathbf{q} _{f}$, Eq. (17) is integrated in time to obtain

18 $$ \mathbf{S} \mathbf{q}_{f} =\mathbf{0}. $$

By applying the mean-axis frame condition (18), the flexible body can no longer undergo rigid body motion with respect to the reference frame. For illustration, the kinematic description of a crank–shaft system for mean-axis frame is also given in Fig. 3. In the mean-axis frame, the relative displacement and rotation (green mesh) of the body exhibit a interface-free vibration with respect to the reference frame (gray mesh). Generally, the relative displacement and rotation observed from a mean-axis frame will be smaller than their counterparts observed from the nodal-fixed frame, as discussed in [19].

### Embedding of mean-axis and interface constraints

While enforcing Eq. (11) for nodal-fixed frame is straightforward, the treatment of Eq. (18) requires more attention, since the constraint conditions are expressed as an explicit form of all relative DoFs $\mathbf{q}_{f}$. By noticing that the mean-axis frame only yields linear constraints, we apply the so-called *embedding technique* [5] to obtain a minimum number of equations expressed in terms of independent coordinates. As mentioned in [27], the process of imposing all the reference conditions is actually equivalent to static condensation, where the slave variables are eliminated.

We can define the generalized DoFs vector $\mathbf{q}_{g}$ as

19 $$ \mathbf{q}_{g}=\operatorname{col}(\mathbf{q}_{v},\mathbf{q}_{b},\mathbf{q}_{i}) =\operatorname{col}( \mathbf{q}_{m,v} ,\mathbf{q}_{s,b}, \mathbf{q}_{m,i} , \mathbf{q}_{s,i}) $$

where the virtual, boundary and internal DoFs are further split into independent (master) and dependent (slave) sets of coordinates, denoted by the subscript $\star _{m}$ and $\star _{s}$, respectively. Note that all interface DoFs $\mathbf{q}_{b}$ are set as slave DoFs $\mathbf{q} _{s,b}$ and the DoFs of the virtual nodes are set as master DoFs $\mathbf{q}_{m,v}$ since $\mathbf{q}_{s,b}$ are determined by $\mathbf{q}_{m,v}$.

The rigid interface condition in (10) and mean-axis frame constraint in (18) can be written together as

20 $$ \underbrace{ \begin{bmatrix} \mathbf{L}_{v} & -\mathbf{I} & \mathbf{0} &\mathbf{0}\\ \mathbf{0} & \mathbf{S}_{s,b} & \mathbf{S}_{m,i} & \mathbf{S}_{s,i} \end{bmatrix} } _{\mathbf{D}} \underbrace{ \begin{bmatrix} \mathbf{q}_{m,v}\\ \mathbf{q}_{s,b}\\ \mathbf{q}_{m,i}\\ \mathbf{q}_{s,i} \end{bmatrix} } _{\mathbf{q}_{g}} = \mathbf{0}, $$

where $\mathbf{D}\in \mathbb{R}^{(n_{b}+6)\times (n_{v}+n_{b}+n_{i})}$ is the Jacobian matrix of all constraint conditions with respect to the generalized DoFs $\mathbf{q}_{g}$ in the mean-axis frame, and the $\mathbf{S}$ matrix has been partitioned accordingly. Equation (20) can also be written as

21 $$ \mathbf{D}_{s}\mathbf{q}_{s}+\mathbf{D}_{m} \mathbf{q}_{m} = \mathbf{0}, $$

where

22 $$ \mathbf{q}_{s}=\operatorname{col}( \mathbf{q}_{s,b} ,\mathbf{q}_{s,i}) \quad \text{and} \quad \mathbf{q}_{m}= \operatorname{col}(\mathbf{q}_{m,v} , \mathbf{q}_{m,i}), $$

and the matrices $\mathbf{D}_{s}$ and $\mathbf{D}_{m}$ contain the columns of $\mathbf{D}$ corresponding to slave DoFs $\mathbf{q}_{s} \in \mathbb{R}^{n_{b}+6}$ and master DoFs $\mathbf{q}_{m} \in \mathbb{R}^{n_{m}}$, respectively, and $n_{m}$ are the number of master DoFs. The generalized DoFs $\mathbf{q}_{g}$ can then be written as a function of $\mathbf{q}_{m}$ as

23 $$ \mathbf{q}_{g} = \begin{bmatrix} \mathbf{q}_{m}\\ \mathbf{q}_{s} \end{bmatrix} = \begin{bmatrix} \mathbf{I}\\ -(\mathbf{D}_{s})^{-1}\mathbf{D}_{m} \end{bmatrix} \mathbf{q}_{m} = \mathbf{H}_{m} \mathbf{q}_{m}, $$

where $\mathbf{H}_{m}$ is the generalized condensation matrix. Finally, according to (23), the relative DoFs $\mathbf{q} _{f}$ can be directly written as a function of the master DoFs $\mathbf{q}_{m}$ as

24 $$ \mathbf{q}_{f} = \mathbf{H}_{fm} \mathbf{q}_{m}, $$

where $\mathbf{H}_{fm}$ contains the rows of $\mathbf{H}_{m}$ corresponding to $\mathbf{q}_{f}$.

By substituting (24) into (8) and performing a Galerkin projection, we can obtain the EoMs as

25 $$ \underbrace{ \begin{bmatrix} \mathbf{M}_{RR} & \mathbf{M}_{R\theta } & \mathbf{M}_{Rm}\\ & \mathbf{M}_{\theta \theta } & \mathbf{M}_{\theta m}\\ \text{sym} & & \mathbf{M}_{mm} \end{bmatrix} }_{\widetilde{\mathbf{M}}} \underbrace{ \begin{bmatrix} \ddot{\mathbf{R}}\\ \ddot{\boldsymbol{\theta }}\\ \ddot{\mathbf{q}}_{m} \end{bmatrix} } _{\ddot{\widetilde{\mathbf{q}}}} - \underbrace{ \begin{bmatrix} \mathbf{Q}_{R}\\ \mathbf{Q}_{\theta }\\ \mathbf{Q}_{m} \end{bmatrix} } _{\widetilde{\mathbf{Q}}} + \underbrace{ \begin{bmatrix} \mathbf{0}\\ \mathbf{0}\\ \mathbf{f}_{m} \end{bmatrix} }_{\widetilde{\mathbf{f}}} = \underbrace{ \begin{bmatrix} \mathbf{g}_{R}\\ \mathbf{g}_{\theta }\\ \mathbf{g}_{m} \end{bmatrix} }_{\widetilde{\mathbf{g}}}, $$

where

$$ \begin{aligned} & \mathbf{M}_{mm}=(\mathbf{H}_{fm})^{\text{T}} \mathbf{M}_{ff} \mathbf{H}_{fm}, \qquad \mathbf{M}_{Rm}= \mathbf{M}_{Rf}\mathbf{H}_{fm}, \qquad \mathbf{f}_{m}=( \mathbf{H}_{fm})^{\text{T}}\mathbf{f}_{f} \\ & \mathbf{g}_{m}=(\mathbf{H}_{fm})^{\text{T}} \mathbf{g}_{f}, \qquad \mathbf{M}_{\theta m}=\mathbf{M}_{\theta f} \mathbf{H}_{fm}, \qquad \mathbf{Q}_{m}=( \mathbf{H}_{fm})^{\text{T}}\mathbf{Q}_{f}. \end{aligned} $$

In (25), the EoMs are expressed in terms of only the master DoFs $\mathbf{q}_{m}$. The $\tilde{\star }$ refers to quantities relative to a flexible body constrained on the mean-axis frame. The constraint condition in (20) will be identically satisfied. This procedure is referred as the *embedding technique* in [5]. This embedding technique is not as computationally efficient as using Lagrange multipliers, since the condensed tangent stiffness and mass matrices are characterized by a worse sparsity pattern as compared to the unconstrained, full counterparts. However, it is strongly preferred when applying MOR for the relative DoFs, as any mode extracted from Eq. (25) and used to form the reduction basis would satisfy the mean axis and rigid interface condition exactly.

## Flexible multibody equations

Holonomic joint constraints are applied to connect neighboring bodies and/or impose prescribed motion through virtual nodes. For instance, a rigid connection between $j$th and $k$th bodies can be imposed as

26 $$ \mathbf{C}\{\mathbf{R},\boldsymbol{\theta },\mathbf{q}_{m,v}\}= \mathbf{R}^{(j)}+\mathbf{A}^{(j)}\mathbf{N}_{v}^{(j)} \bigl(\mathbf{q}_{0,v} ^{(j)}+\mathbf{q}^{(j)}_{m,v} \bigr)-\mathbf{R}^{(k)}-\mathbf{A}^{(k)} \mathbf{N}_{v}^{(k)} \bigl(\mathbf{q}_{0,v}^{(k)}+\mathbf{q}^{(k)}_{m,v} \bigr)= \mathbf{0}, $$

where $\mathbf{q}_{0,v}$ and $\mathbf{q}_{m,v}$ are the initial position and relative DoFs of the connecting virtual nodes, respectively, and $\mathbf{N}_{v}$ here is equal to the Boolean matrix of selecting the translation DoFs.

It is emphasized here that the joint constraints are not imposed at the internal DoFs $\mathbf{q}_{m,i}$. The constraint Jacobian matrix can thus be written as

27 $$ {\mathbf{C}}_{q}=\frac{\partial \mathbf{C}}{\partial \widetilde{\mathbf{q}}}= \biggl[ \frac{\partial \mathbf{C}}{\partial \mathbf{R}} \quad \frac{\partial \mathbf{C}}{\partial \boldsymbol{\theta }} \quad \frac{\partial \mathbf{C}}{\partial \mathbf{q}_{m,v}} \quad \mathbf{0}_{i} \biggr] = [ \mathbf{C}_{R} \quad \mathbf{C}_{\theta } \quad \mathbf{C}_{m} ] , $$

where the block $\mathbf{0}_{i}$ reflects the fact that the constraints are not imposed on the internal DoFs $\mathbf{q}_{m,i}$ and $\mathbf{C}_{m}= [ \frac{\partial \mathbf{C}}{\partial \mathbf{q}_{m,v}} \ \mathbf{0}_{i} ] $. The joint constraints (usually nonlinear) are included with Lagrange multipliers $\boldsymbol{\lambda }$ as

28 $$ \left\{ \textstyle\begin{array}{l} \begin{bmatrix} \mathbf{M}^{(s)}_{RR} & \mathbf{M}^{(s)}_{R\theta } & \mathbf{M}^{(s)}_{Rm}\\ & \mathbf{M}^{(s)}_{\theta \theta } & \mathbf{M}^{(s)}_{\theta m}\\ \text{sym} & & \mathbf{M}^{(s)}_{mm} \end{bmatrix} \begin{bmatrix} \ddot{\mathbf{R}}^{(s)}\\ \ddot{\boldsymbol{\theta }}^{(s)}\\ \ddot{\mathbf{q}}^{(s)}_{m} \end{bmatrix} - \begin{bmatrix} \mathbf{Q}^{(s)}_{R}\\ \mathbf{Q}^{(s)}_{\theta }\\ \mathbf{Q}^{(s)}_{m} \end{bmatrix} + \begin{bmatrix} \mathbf{0}\\ \mathbf{0}\\ \mathbf{f}^{(s)}_{m} \end{bmatrix} + \begin{bmatrix} (\mathbf{C}^{(s)}_{R})^{\text{T}}\\ (\mathbf{C}^{(s)}_{\theta })^{\text{T}}\\ (\mathbf{C}^{(s)}_{m})^{\text{T}} \end{bmatrix} \boldsymbol{\lambda } = \begin{bmatrix} \mathbf{g}^{(s)}_{R}\\ \mathbf{g}^{(s)}_{\theta }\\ \mathbf{g}^{(s)}_{m} \end{bmatrix}\\ \quad\text{for } s=1,\dots , \mathcal{H},\\ \mathbf{C}\{\mathbf{R}, \boldsymbol{\theta }, \mathbf{q}_{m,v}\}=\mathbf{0}, \end{array}\displaystyle \right. $$

where ℋ is the number of bodies in the FMBS.

## Enhanced Rubin substructuring method

The inertial terms of (28) are configuration dependent and therefore need to be updated at every time step during time integration. Likewise, when geometric nonlinearities have to be considered, also the internal force vector $\mathbf{f}_{m}$ is configuration dependent. The computational cost of large size nonlinear FMBS using the FFR reference may thus become significant, and MOR is required. The idea is to reduce the size of $\mathbf{q}_{m}$ for each body by expressing them as a combination of modes computed after suppressing the rigid body motion of the floating frame, see [1, 5, 28]. The EoMs for each body in (28) can thus be simplified by suppressing $\operatorname{col}(\mathbf{R},\boldsymbol{\theta })$ as

29 $$ \mathbf{M}_{mm}\ddot{\mathbf{q}}_{m} + \mathbf{f}_{m}\{\mathbf{q}_{m} \} = \mathbf{g}_{m}- \mathbf{C}_{m}^{\text{T}}\boldsymbol{\lambda }. $$

By fixing the rigid body motion of the frame, the quadratic velocity terms $\mathbf{Q}_{m}$ and the couplings between rigid body motion and relative displacement vanish. The last term in (29) represents the connecting forces which are imposed only at the virtual node DoFs. The last term can be further rewritten as

30 $$ -\mathbf{C}^{\text{T}}_{m}\boldsymbol{\lambda }= \mathbf{B}^{\text{T}} \mathbf{g}_{v}, $$

where $\mathbf{B} \in \mathbb{R}^{n_{v} \times n_{m}}$ is the local-Boolean matrix that selects the interface DoFs $\mathbf{q}_{v}$ from $\mathbf{q}_{m}$, and $\mathbf{g}_{v} \in \mathbb{R}^{n_{v}}$ is the interface force imposed at the virtual node.

Furthermore, we linearized (29) as

31 $$ \mathbf{M}_{mm}\ddot{\mathbf{q}}_{m} + \mathbf{K}_{mm}\mathbf{q}_{m} = \mathbf{g}_{m}+ \mathbf{B}^{\text{T}}\mathbf{g}_{v}, $$

where

$$ \mathbf{K}_{mm}= \frac{\mathrm{d} \mathbf{f}_{m}}{\mathrm{d} \mathbf{q}_{m}} \bigg|_{\mathbf{0}} =(\mathbf{H}_{fm})^{\text{T}} \frac{\mathrm{d} \mathbf{f}_{f}}{\mathrm{d} \mathbf{q}_{f}} \bigg|_{\mathbf{0}}\frac{\mathrm{d}\mathbf{q}_{f}}{ \mathrm{d}\mathbf{q}_{m}} =(\mathbf{H}_{fm})^{\text{T}} \mathbf{K}_{ff} \mathbf{H}_{fm} $$

is the linear stiffness matrix after the constraint embedding, and $\mathbf{K}_{ff}$ is the linear sparse stiffness matrix of the body. In general, $\mathbf{K}_{mm}$ features decreased sparsity patterns as compared to $\mathbf{K}_{ff}$.

### Augmented Rubin reduction bases with modal derivatives

In this section, we extend the standard Rubin substructuring method by augmenting the associated reduction basis with MDs to properly consider geometric nonlinear effects. The ROBs are established for each body separately.

The MDs were first proposed in [17, 18] for a single structure not undergoing rigid body motion, by differentiating the eigenvalue problem associated to the free vibration with respect to the modal amplitude. The computation of MDs is discussed in detail in [30]. The methods in [30] require an explicit form of the internal nonlinear forces. Alternatively, Slaats [29] proposed the use of finite difference, which allows the computation of the MDs by means of standard nonlinear FE programs, as this method does not require access to the nonlinear forces and Jacobians. Related to this property, we applied the simplified definition of MDs by neglecting these inertia related terms. This technique is usually addressed as the *definition without mass consideration*, or more recently, *static* MDs [30]. A more theoretical grounding of the validity of MDs is given in [31].

When the inertial terms are neglected, Eq. (29) becomes

32 $$ \mathbf{f}_{m} \{\mathbf{q}_{m}\} = \mathbf{g}_{m} +\mathbf{B}^{ \text{T}}\mathbf{g}_{v}. $$

We assume here that the external load $\mathbf{g}_{m}$ can be written as a stiffness-scaled linear superposition of the free-interface modes (FVMs) as

33 $$ \mathbf{g}_{m}=\mathbf{K}_{mm}\boldsymbol{ \varPhi }\boldsymbol{\eta }, $$

where the FVMs $\boldsymbol{\varPhi }$ can be obtained by solving the linear eigenvalue problem associated to (31):

34 $$ \bigl( \mathbf{K}_{mm}-\nu ^{2}_{j} \mathbf{M}_{mm} \bigr) \boldsymbol{\phi }_{j}=\mathbf{0}, $$

where $\nu _{j}$ is the $j$th eigenfrequency and $\boldsymbol{\phi } _{j}$ is the corresponding FVM. Generally, a truncated set of the first $r_{m}$ FVMs is selected in the reduction basis $\boldsymbol{\varPhi } \in \mathbb{R}^{n_{m} \times r_{m}}$ based on the frequency range of interest. The reduction will be achieved by letting $r_{m}\ll n_{m}$. Note that $\boldsymbol{\varPhi }$ does not contain any rigid body motion since the system has already been fully constrained, see Sect. 3.

By substituting (33) into (32), we obtain a static nonlinear problem

35 $$ \mathbf{f}_{m}\{\mathbf{q}_{m}\}= \mathbf{K}_{mm}\boldsymbol{\varPhi } \boldsymbol{\eta }+ \mathbf{B}^{\text{T}}\mathbf{g}_{v} = \begin{bmatrix} \mathbf{K}_{mm}\boldsymbol{\varPhi } & \mathbf{B}^{\text{T}} \end{bmatrix} \boldsymbol{\zeta }, \quad \text{with } \boldsymbol{\zeta }= \operatorname{col}(\boldsymbol{\eta }, \mathbf{g}_{v}), $$

where static response $\mathbf{q}_{m}$ is determined by the modal amplitude $\boldsymbol{\zeta }\in \mathbb{R}^{r_{m}+n_{v}}$. Instead of finding a solution of (35), we assume that $\mathbf{q}_{m}$ is $C^{2}$ differentiable with respect to the modal parameter $\boldsymbol{\zeta }$ and we expand $\mathbf{q}_{m}$ into a Taylor series around equilibrium position as

36 $$ \mathbf{q}_{m}\{\boldsymbol{\zeta }\}=\sum _{j=1}^{r_{m}+n_{v}} \frac{\partial \boldsymbol{\mathbf{q}}_{m}}{\partial \zeta _{j}} \bigg|_{\mathbf{0}}\zeta _{j}+\frac{1}{2}\sum _{j=1}^{r_{m}+n_{v}}\sum_{k=1} ^{r_{m}+n_{v}} \frac{\partial ^{2} \boldsymbol{\mathbf{q}}_{m}}{ \partial \zeta _{j} \partial \zeta _{k}} \bigg| _{\mathbf{0}}\zeta _{j} \zeta _{k}+\mathcal{O}\bigl(\|\boldsymbol{\zeta}\|^{3}\bigr), $$

where $\zeta _{j}$ is the $j$th component of the modal parameter $\boldsymbol{\zeta }$.

In order to find the derivatives in (36) we differentiate both sides of (35) with respect to $\boldsymbol{\zeta }$, and evaluate them around the equilibrium position as

37 $$ \frac{\mathrm{d} \mathbf{f}_{m}}{\mathrm{d} \mathbf{q}_{m}}\frac{ \partial \mathbf{q}_{m}}{\partial \boldsymbol{\zeta }}= \begin{bmatrix} \mathbf{K}_{mm}\boldsymbol{\varPhi } & \mathbf{B}^{\text{T}} \end{bmatrix} \quad \rightarrow \quad \frac{\partial \mathbf{q}_{m}}{\partial \boldsymbol{\zeta }} \bigg|_{\mathbf{0}}= \begin{bmatrix} \boldsymbol{\varPhi } & \mathbf{K}_{mm}^{-1}\mathbf{B}^{\text{T}} \end{bmatrix} , $$

where $\mathbf{K}_{mm}^{-1}$ exists as rigid body motions are suppressed. This procedure distinguishes from the standard Rubin method, where a pseudo-inverse matrix needs to be computed due to the presence of rigid body modes. The matrix $\boldsymbol{\varPsi }=\mathbf{K}_{mm} ^{-1}\mathbf{B}^{\text{T}}$, $\boldsymbol{\varPsi }\in \mathbb{R}^{n _{m}\times n_{v}}$ includes the so-called attachment modes (AMs). The AMs represent deformations due to the unit generalized force at one interface DoF and zero to all other interface DoFs. Therefore, the connecting interface force vector $\mathbf{g}_{v}$ represents the modal amplitudes of the AMs $\boldsymbol{\varPsi }$. Expression (37) can be compactly written as

38 $$ \frac{\partial \mathbf{q}_{m}}{\partial \boldsymbol{\zeta }} \bigg|_{\mathbf{0}}= \begin{bmatrix} \boldsymbol{\varPhi } & \boldsymbol{\varPsi } \end{bmatrix} = \mathbf{X}, $$

where $\mathbf{X}$ is the standard Rubin ROB. In order to calculate the second-order derivatives of $\mathbf{q}_{m}$ with respect to $\boldsymbol{\zeta }$, we differentiate (35) twice to get

39 $$ \frac{\mathrm{d} \mathbf{f}_{m}}{\mathrm{d} \mathbf{q}_{m}}\frac{ \partial ^{2} \mathbf{q}_{m}}{\partial \zeta _{j} \partial \zeta _{k}} + \frac{\mathrm{d}^{2} \mathbf{f}_{m}}{\mathrm{d} (\mathbf{q}_{m})^{2}} \frac{ \partial \mathbf{q}_{m}}{\partial \zeta _{j}}\frac{\partial \mathbf{q} _{m}}{\partial \zeta _{k}} = \mathbf{0}. $$

Evaluating (39) around the equilibrium position gives

40 $$ \mathbf{K}_{mm} \frac{\partial ^{2} \mathbf{q}_{m}}{\partial \zeta _{j} \partial \zeta _{k}} \bigg| _{\mathbf{0}} + \frac{ \mathrm{d}^{2} \mathbf{f}_{m}}{\mathrm{d} (\mathbf{q}_{m})^{2}} \bigg| _{\mathbf{0}} \frac{\partial \mathbf{q}_{m}}{\partial \zeta _{j}} \bigg| _{\mathbf{0}} \frac{\partial \mathbf{q}_{m}}{ \partial \zeta _{k}} \bigg| _{\mathbf{0}} = \mathbf{0}, $$

where the right-hand side of (40) is a null vector, since the external load and interface forces are assumed to be a linear superposition of the modal parameters $\boldsymbol{\zeta }$. The second derivatives of the nonlinear response with respect to the modal amplitudes $\frac{\partial ^{2} \mathbf{q}_{m}}{\partial \zeta _{j} \partial \zeta _{k}} \big|_{\mathbf{0}}$ are the MDs, computed from (40) as

41 $$ \boldsymbol{\vartheta }_{jk} = \frac{\partial ^{2} \mathbf{q} _{m}}{\partial \zeta _{j} \partial \zeta _{k}} \bigg| _{\mathbf{0}} = -( \mathbf{K}_{mm})^{-1} \frac{\mathrm{d}^{2} \mathbf{f}_{m}}{ \mathrm{d} (\mathbf{q}_{m})^{2}} \bigg|_{\mathbf{0}}\mathbf{X}_{j} \mathbf{X}_{k}, $$

and it holds that $\boldsymbol{\vartheta }_{jk}= \boldsymbol{\vartheta }_{kj}$, see [23]. Note that $\frac{\mathrm{d}^{2} \mathbf{f}_{m}}{\mathrm{d} (\mathbf{q} _{m})^{2}} \big|_{\mathbf{0}}\mathbf{X}_{j}$ is the directional derivative of internal tangent stiffness matrix $\overline{\mathbf{K}}_{mm}$ with respect to the modal amplitudes $\zeta _{j}$ of mode $\mathbf{X}_{j}$, i.e.,

42 $$ \frac{\mathrm{d}^{2} \mathbf{f}_{m}}{\mathrm{d} (\mathbf{q} _{m})^{2}} \bigg| _{\mathbf{0}} \mathbf{X}_{j} = \frac{ \mathrm{d} \overline{\mathbf{K}}_{mm}}{\mathrm{d} \mathbf{q}_{m}} \bigg|_{0} \cdot \frac{\partial \mathbf{q} _{m}}{\partial \zeta _{j}} \bigg|_{0} = \frac{\partial \overline{ \mathbf{K}}_{mm}}{\partial \zeta _{j}} \bigg|_{0} = \lim_{ \zeta _{j} \to 0} \frac{1}{\zeta _{j}} \bigl[ \overline{ \mathbf{K}}_{mm} \{ \mathbf{X}_{j} \zeta _{j} \} -\overline{ \mathbf{K}}_{mm} \{ \mathbf{0} \} \bigr] . $$

Equation (42) certifies that MDs can be computed numerically by the finite difference method, as proposed by [29]. This numerical approach recomputes the configuration dependent stiffness matrix when the structure perturbed from the equilibrium position in the direction of one mode. The perturbation should be small enough for the accuracy of finite difference, but it cannot be too small in order not to incur in round-off errors.

In this paper, the von Kármán kinematic model is applied. Since the internal force vector and stiffness matrix can be explicitly expressed as a polynomial function of the DoFs, the MDs can be computed analytically.

Having defined the AMs, FVMs and corresponding MDs, $\mathbf{q}_{m}$ can be approximated by the second-order Taylor expansion in (36) as

43 $$ \mathbf{q}_{m} = \sum_{j=1}^{r_{m}+n_{v}} \mathbf{X}_{j}\zeta _{j}+ \frac{1}{2}\sum _{j=1}^{r_{m}+n_{v}}\sum_{k=1}^{r_{m}+n_{v}} \boldsymbol{\vartheta }_{jk}\zeta _{j}\zeta _{k}, $$

which constitutes a quadratic manifold for $\mathbf{q}_{m}$ in the $\boldsymbol{\zeta }$ space. In this work, we will not directly apply (43), as done, for instance, in [30, 32]. Instead, the MDs will be included in the ROB as additional independent modes to reproduce geometric nonlinearities.^1^

The relative DoFs vector $\mathbf{q}_{m}$ with respect to the floating frame is then given by

44 $$ \mathbf{q}_{m} = \boldsymbol{\varPsi } \mathbf{g}_{v}+\boldsymbol{\varPhi } \boldsymbol{\eta } + \boldsymbol{ \varTheta }\boldsymbol{\xi }, $$

where $\boldsymbol{\varTheta }$ is the matrix containing the vectors of independent MDs, and $\boldsymbol{\xi }$ is the modal coordinates vector associated to the MDs in $\boldsymbol{\varTheta }$. Since the MDs are calculated around the equilibrium position and will not be updated during the time integration, the accuracy of using the modal transformation in (44) will be determined by how far the structure departs from reference position.

**Fig. 4:**
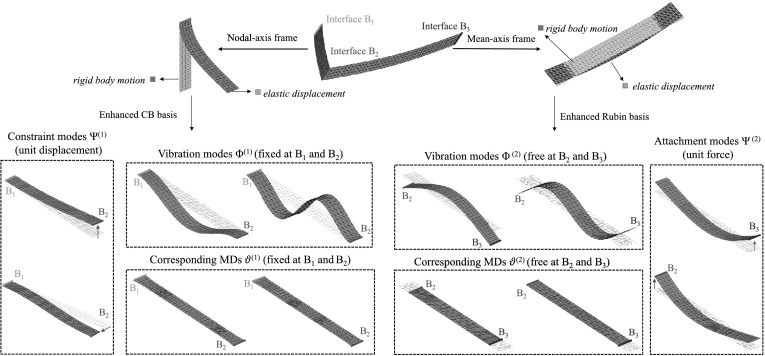
The nonlinear ROBs for a crank–shaft system. The first body (left) and its corresponding enhanced CB ROB is illustrated using the nodal-fixed frame, while the second body (right) and its corresponding enhanced Rubin ROB is illustrated using the mean-axis frame

For illustration, some representative modes of the ROB for a crank–shaft system are shown in Fig. 4. For the first body, the ROB is constructed as done in [1], i.e., a nodal-fixed frame is applied, and the enhanced CB method is applied. The origin of the reference frame is attached to the interface $B_{1}$. Therefore, the fixed-interface modes $\boldsymbol{\varPhi }^{(1)}$, MDs $\boldsymbol{\varTheta }^{(1)}$ are fixed at interface $B_{1}$ and $B_{2}$. The compatibility with neighboring bodies is considered by the constraint modes (CMs) $\boldsymbol{\varPsi }^{(1)}$. The mean-axis frame is utilized to describe the motion of the second body. The corresponding FVMs $\boldsymbol{\varPhi }^{(2)}$, MDs $\boldsymbol{\varTheta }^{(2)}$ and AMs $\boldsymbol{\varPsi }^{(2)}$ exhibit free motion at the interface sets $B_{2}$ and $B_{3}$. It should be noted that all modes are obtained after the mean-axis constraints are included. Therefore, the FVMs in the ROB of the mean-axis frame model contain no rigid body motions. While these low-frequency FVMs of the flat plate models contain bending-dominant vibrations, the corresponding MDs exhibit membrane-dominant vibrations to properly model the nonlinear effects.

In order to assemble the reduced components, Eq. (44) should contain only modal amplitudes. A further transformation is applied to replace the force vector $\mathbf{g}_{v}$ with the interface DoFs vector $\mathbf{q}_{m,v}$. The interface partition of (44) is

45 $$ \mathbf{q}_{m,v}=\mathbf{B}\mathbf{q}_{m} = \boldsymbol{ \varPsi }_{v} \mathbf{g}_{v}+\boldsymbol{\varPhi }_{v} \boldsymbol{\eta } + \boldsymbol{\varTheta }_{v}\boldsymbol{\xi }, $$

where $\boldsymbol{\varPsi }_{v}$, $\boldsymbol{\varPhi }_{v}$ and $\boldsymbol{\varTheta }_{v}$ are the rows of the AMs, FVMs and MDs associated to the interface DoFs, respectively. The interface force vector $\mathbf{g}_{v}$ can thus be expressed as

46 $$ \mathbf{g}_{v}= (\boldsymbol{\varPsi }_{v})^{-1}( \mathbf{q}_{m,v}- \boldsymbol{\varPhi }_{v}\boldsymbol{\eta }- \boldsymbol{\varTheta }_{v} \boldsymbol{\xi }). $$

By substituting (46) into (44) and recalling (22), the interface DoFs $\mathbf{q}_{m,v}$ can be retained in the final coordinates transformation as

47 $$ \mathbf{q}_{m}= \begin{bmatrix} \mathbf{q}_{m,v}\\ \mathbf{q}_{m,i} \end{bmatrix} = \begin{bmatrix} \mathbf{I} & \mathbf{0} & \mathbf{0}\\ \boldsymbol{\varPsi }_{iv} & \boldsymbol{\varPhi }_{i}-\boldsymbol{\varPsi } _{iv}\boldsymbol{\varPhi }_{v} & \boldsymbol{\varTheta }_{i}- \boldsymbol{\varPsi }_{iv}\boldsymbol{\varTheta }_{v} \end{bmatrix} \begin{bmatrix} \mathbf{q}_{m,v}\\ \boldsymbol{\eta }\\ \boldsymbol{\xi } \end{bmatrix} = \mathbf{V}_{f}\boldsymbol{\gamma }_{f}, $$

where $\boldsymbol{\varPsi }_{iv}=\boldsymbol{\varPsi }_{i}( \boldsymbol{\varPsi }_{v})^{-1}$, $\boldsymbol{\varPsi }_{i}$, $\boldsymbol{\varPhi }_{i}$ and $\boldsymbol{\varTheta }_{i}$ are the internal components of the AMs, FVMs and MDs, respectively. $\mathbf{V}_{f}$ and $\boldsymbol{\gamma }_{f}$ are the final ROB and generalized DoFs vector for the enhanced Rubin reduction, when geometric nonlinearity is considered.

### Reduced equation of motion

The final reduced EoMs for the $s$th substructure can be obtained by substituting (47) into (28) and performing a Galerkin projection as

48 $$ \underbrace{ \begin{bmatrix} \mathbf{M}^{(s)}_{RR} & \mathbf{M}^{(s)}_{R\theta } & \overline{\mathbf{M}}^{(s)}_{Rf}\\[4pt] & \mathbf{M}^{(s)}_{\theta \theta } & \overline{\mathbf{M}}^{(s)}_{\theta f}\\[4pt] \text{sym} & & \overline{\mathbf{M}}^{(s)}_{ff} \end{bmatrix} } _{\overline{\mathbf{M}}^{(s)}} \underbrace{ \begin{bmatrix} \ddot{\mathbf{R}}^{(s)} \\ \ddot{\boldsymbol{\theta }}^{(s)} \\ \ddot{\boldsymbol{\gamma }}^{(s)}_{f} \end{bmatrix} } _{\ddot{\overline{\mathbf{q}}}^{(s)}} - \underbrace{ \begin{bmatrix} \mathbf{Q}^{(s)}_{R} \\[4pt] \mathbf{Q}^{(s)}_{\theta } \\[4pt] \overline{\mathbf{Q}}^{(s)}_{f} \end{bmatrix} } _{\overline{\mathbf{Q}}^{(s)}} + \underbrace{ \begin{bmatrix} \mathbf{0} \\ \mathbf{0} \\ \overline{\mathbf{f}}^{(s)}_{f} \end{bmatrix} }_{\overline{\mathbf{f}}^{(s)}} + \underbrace{ \begin{bmatrix} (\mathbf{C}^{(s)}_{R})^{\text{T}} \\[4pt] (\mathbf{C}^{(s)}_{\theta })^{\text{T}} \\[4pt] (\overline{\mathbf{C}}^{(s)}_{f})^{\text{T}} \end{bmatrix} } _{(\overline{\mathbf{C}}^{(s)})^{\text{T}}} \boldsymbol{\lambda } = \underbrace{ \begin{bmatrix} \mathbf{g}^{(s)}_{R} \\[4pt] \mathbf{g}^{(s)}_{\theta } \\[4pt] \overline{\mathbf{g}}^{(s)}_{f} \end{bmatrix} } _{\overline{\mathbf{g}}^{(s)}} $$

with

$$ \begin{aligned} & \overline{\mathbf{M}}^{(s)}_{ff}= \bigl(\mathbf{V}^{(s)}_{f}\bigr)^{\text{T}} \mathbf{M}^{(s)}_{mm}\mathbf{V}^{(s)}_{f}, \qquad \overline{\mathbf{M}}^{(s)}_{Rf}=\mathbf{M}^{(s)}_{Rm} \mathbf{V}^{(s)} _{f}, \qquad \overline{\mathbf{C}}^{(s)}_{f}= \mathbf{C}^{(s)}_{m} \mathbf{V}^{(s)}_{f}, \qquad \overline{\mathbf{f}}^{(s)}_{f}=\bigl( \mathbf{V}^{(s)}_{f}\bigr)^{\text{T}}\mathbf{f}^{(s)}_{m} \\ & \overline{\mathbf{g}}^{(s)}_{f}=\bigl(\mathbf{V}^{(s)}_{f} \bigr)^{\text{T}} \mathbf{g}^{(s)}_{m}, \qquad \overline{ \mathbf{M}}^{(s)}_{\theta f}= \mathbf{M}^{(s)}_{\theta m} \mathbf{V}^{(s)}_{f}, \qquad \overline{ \mathbf{Q}}^{(s)}_{f}= \bigl(\mathbf{V}^{(s)}_{f}\bigr)^{\text{T}} \mathbf{Q}^{(s)} _{m}. \end{aligned} $$

All nonlinear terms in (48) can be directly expressed as a function of the modal coordinates $\operatorname{col}(\mathbf{R}^{(s)}, \boldsymbol{\theta }^{(s)}, \boldsymbol{\gamma }^{(s)}_{f})$ by a tensorial form as in [1].

The nonlinear force vector $\overline{\mathbf{f}}^{(s)}_{f}$, whose update is the most time consuming operation during each iteration of the time integration, can be directly expressed as

49 $$ \overline{\mathbf{f}}^{(s)}_{f}={^{2} \mathcal{W}^{(s)}} \boldsymbol{\gamma }^{(s)}_{f}+ \bigl( {^{3}\mathcal{W}^{(s)}}\cdot \boldsymbol{\gamma }^{(s)}_{f} \bigr) \cdot \boldsymbol{\gamma }^{(s)} _{f}+ \bigl[ \bigl( {^{4}\mathcal{W}^{(s)}}\cdot \boldsymbol{\gamma }^{(s)}_{f} \bigr) \cdot \boldsymbol{\gamma }^{(s)} _{f} \bigr] \cdot \boldsymbol{\gamma }^{(s)}_{f}, $$

where ${^{2}\mathcal{W}^{(s)} \in \mathbb{R}^{r^{(s)}_{g}\times r^{(s)} _{g}}}$, ${^{3}\mathcal{W}^{(s)} \in \mathbb{R}^{r^{(s)}_{g}\times r ^{(s)}_{g}\times r^{(s)}_{g}}}$ and ${^{4}\mathcal{W}^{(s)} \in \mathbb{R}^{r^{(s)}_{g}\times r^{(s)}_{g}\times r^{(s)}_{g}\times r ^{(s)}_{g}}}$ are constant quadratic, cubic and quartic tensors, respectively, and $r^{(s)}_{g}$ is the number of modes in the enhanced ROB $\mathbf{V}_{f}^{(s)}$. The tensors ${^{2}\mathcal{W}^{(s)}}$, ${^{3}\mathcal{W}^{(s)}}$ and ${^{4}\mathcal{W}^{(s)}}$ can be calculated *offline*, once the reduction basis of each flexible body is determined.

The system reduced EoMs can be obtained by assembling the contribution from each body and by appending the constraint conditions as

50 $$ \left\{ \textstyle\begin{array}{l@{\quad}l} \overline{\mathbf{M}}^{(s)}\ddot{\overline{\mathbf{q}}}^{(s)}-\overline{ \mathbf{Q}}^{(s)}+\overline{\mathbf{f}}^{(s)}+(\overline{\mathbf{C}} ^{(s)})^{\text{T}}\boldsymbol{\lambda }=\overline{\mathbf{g}}^{(s)} , &\text{for } s=1, \dots , \mathcal{H},\\ \mathbf{C}\{\mathbf{R}, \boldsymbol{\theta }, \mathbf{q}_{m,v}\}= \mathbf{0}. \end{array}\displaystyle \right. $$

It is emphasized here that the constraint equation $\mathbf{C}= \mathbf{0}$ is not projected onto the reduced basis, and therefore, exact interface compatibility has been guaranteed.

In this work, we use the implicit Newmark scheme for the time integration of (50) by setting the integration parameters $\gamma =\frac{1}{2}$ and $\beta =\frac{1}{4}$. The artificial damping coefficient $\alpha $ is set to zero for all the presented examples. The constraint equation is treated as discussed in [33], where the Lagrange multipliers have been set as additional DoFs. Substantial computational cost reduction can be achieved, in comparison to full analysis, thanks to the reduction in size and the efficient treatment of the nonlinear terms (49). The computational efficiency of applying the implicit Newmark scheme has been discussed in [1], and will not be repeated here.

## Numerical examples

In this section, two numerical examples are presented to assess the performance of the proposed reduction method. All the models contain elastic bodies meshed with triangular FE shell elements [34] featuring 3 nodes per element and 6 DoFs per node. The von Kármán kinematic model is adopted. The detailed formulation of the shell element can be found in [34]. In our discussion, the following labeling is utilized to refer to the solutions obtained from different approaches, namely:

- MFR-HFM-L/NL: Linear/Nonlinear response of the High Fidelity Model (HFM) obtained from Mean-axis floating Frame of Reference (MFR);
- NFR-HFM-L/NL: Linear/Nonlinear response of the HFM obtained by Nodal-fixed floating Frame of Reference (NFR);
- MFR-ERubin-NL: Nonlinear response of ROMs obtained by projection on the Enhanced Rubin basis (with MDs) for MFR, as discussed in this work;
- NFR-ECB-NL: Nonlinear response of the ROMs obtained by projection on the Enhanced Craig–Bampton basis (with MDs) for NFR, as discussed in [1];
- MFR-Rubin-NL: Nonlinear response of ROMs obtained by the projection on the standard Rubin basis (without MDs) for MFR;
- NFR-CB-NL: Nonlinear response of ROMs obtained by the projection on the standard CB basis (without MDs) for NFR.

### Model 1: rotating beam

**Fig. 5:**
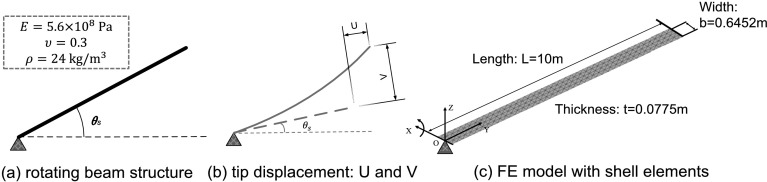
The kinetic description of rotating beam model with shell elements

We consider here the dynamic analysis of a rotating beam, which has been used as a benchmark in many papers dealing with flexible beams and geometric nonlinearities [35–38]. In all the previous publications, the system shown in Fig. 5 was meshed with planar beam elements. The geometry of the beam and material properties are also illustrated in Fig. 5. An imposed end rotation $\theta _{s}$ with respect to $OX$ axis is applied as

$$ \theta _{s} = \left\{ \textstyle\begin{array}{l@{\quad}l} \frac{\omega _{s}}{T_{s}} [\frac{1}{2}t^{2}+ (\frac{T_{s}}{2 \pi } )^{2} (\cos (\frac{2\pi t}{T_{s}} )-1 ) ], & t\leq T_{s},\\ \omega _{s} ( t-\frac{T_{s}}{2} ), & t>T_{s}, \end{array}\displaystyle \right. $$

where $T_{s}=15$ s and $\omega _{s}= 6~\mbox{rad/s}$.

The nonlinear responses of the tip displacement components $W$, $U$ and $V$ (see Fig. 5(b)) obtained with different methods are compared. When the global position vector at the tip node $\mathbf{r}^{j}$ is obtained from Eq. (1), the tip displacement $W$, $U$ and $V$ can be calculated for this example as

51 $$ \begin{bmatrix} W\\ U\\ V \end{bmatrix} = \begin{bmatrix} 1 & 0 & 0\\ 0 & \cos \theta _{s} & -\sin \theta _{s}\\ 0 & \sin \theta _{s} & \cos \theta _{s} \end{bmatrix} ^{\text{T}} \left( \begin{bmatrix} r^{t}_{X}\\ r^{t}_{Y}\\ r^{t}_{Z} \end{bmatrix} - \begin{bmatrix} \widetilde{R}^{t}_{X} \\ \widetilde{R}^{t}_{Y} \\ \widetilde{R}^{t}_{Z} \end{bmatrix} \right) - \begin{bmatrix} \mathbf{q}^{t}_{0,X} \\ \mathbf{q}^{t}_{0,Y} \\ \mathbf{q}^{t}_{0,Z} \end{bmatrix} , \ \ \ \ \text{with } \begin{bmatrix} \mathbf{q}^{t}_{0,X} \\ \mathbf{q}^{t}_{0,Y} \\ \mathbf{q}^{t}_{0,Z} \end{bmatrix} = \begin{bmatrix} 0 \\ 10 \\ 0 \end{bmatrix} . $$

The vector $\widetilde{\mathbf{R}}$ presents the position of virtual node of the joint, and the superscript $t$ denotes the tip node with nodal position $\operatorname{col}(0,10,0)$ at the initial state. Notice that since the beam only rotates about the $x$-axis, it holds that $W=0$.

**Fig. 6:**
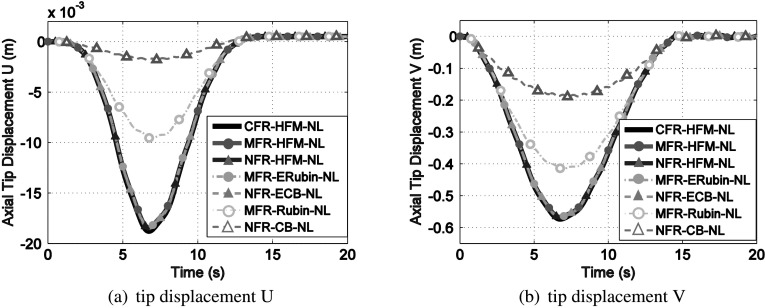
Time history of the tip displacement of the rotating beam. The ROMs featuring MDs (i.e., MFR-ERubin-NL and NFR-ECB-NL) yield the accurate approximations of their corresponding HFM solutions

The tip displacement components $U$ and $V$ are shown in Fig. 6. The nonlinear response obtained from the corotational frame of reference (CFR) featuring planar beam element is also included as a reference solution. The CFR [39] is a more general and expensive framework that is able to deal with arbitrary large elastic displacement. This reference solution is denoted as CFR-HFM-NL. From Fig. 6 we can observe a good agreement between the time response of the CFR-HFM-NL, NFR-HFM-NL and MFR-HFM-NL. The good agreement confirms that the adopted von Kármán kinematic assumption is adequate for this numerical example.

In addition, the accuracy of all ROMs is compared to the HFMs. For illustration, the number of modes for different ROMs is listed in Table 1. Since the origin of the NFR is fixed at the hinge, no interface modes (i.e., constraint modes) are included in the NFR-ECB-NL. The MDs enhanced substructuring method for both nodal-fixed frame and mean-axis frame (i.e., NFR-ECB-NL and MFR-ERubin-NL) can achieve a good approximation of the reference solution. On the contrary, the standard substructuring techniques (without MDs) for both nodal-fixed and mean-axis frame (i.e., NFR-CB-NL and MFR-Rubin-NL) fail to reproduce the full response, even though as many as 50 modes are included in the ROBs.

**Table 1:** Size of ROB for the rotating beam model

	Number of modes in linear ROB	Number of MDs	Total DoFs
NFR-ECB-NL	10	10	20
MFR-ERubin-NL	10	10	20
NFR-CB-NL	50	0	50
MFR-Rubin-NL	50	0	50

**Fig. 7:**
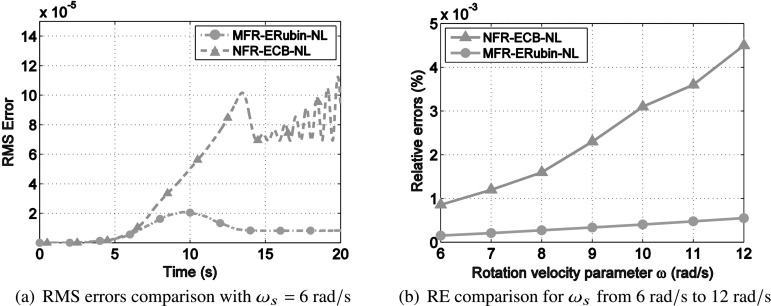
Accuracy comparison between nodal-fixed frame and mean-axis frame. The REs obtained from MFR-ERubin-NL are always significantly lower than their counterparts in NFR-ECB-NL for all the load cases

To further compare the effectiveness of the ROMs between nodal-fixed frame and mean-axis frame, the root-mean square (RMS) error, defined as

52 $$ \epsilon _{RMS}\{t_{i}\}=\sqrt{\frac{1}{n} \bigl( \bigl\| {\mathbf{r}_{X} \{t_{i}\}-\overline{\mathbf{r}}_{X}\{t_{i}\}}\bigr\| ^{2}+\bigl\| { \mathbf{r}_{Y} \{t_{i}\}-\overline{\mathbf{r}}_{Y} \{t_{i}\}}\bigr\| ^{2}+\bigl\| {\mathbf{r}_{Z} \{t_{i}\}-\overline{\mathbf{r}}_{Z}\{t_{i} \}}\bigr\| ^{2} \bigr) }, $$

is plotted in Fig. 7(a) for $\omega _{s}=6~\text{rad}/\text{s}$, and the relative error (RE), defined as

53 $$ RE=\frac{\sqrt{\sum_{t}(\mathbf{r}\{t\}-\overline{\mathbf{r}}\{t\})^{ \text{T}}(\mathbf{r}\{t\}-\overline{\mathbf{r}}\{t\})}}{\sqrt{\sum_{t}\mathbf{r}^{\text{T}}\{t\}\mathbf{r}\{t\}}}\times 100\% , $$

is plotted in Fig. 7(b) for $\omega _{s}$ ranging from 6 to $12~\text{rad}/\text{s}$. Here, $\mathbf{r}$ and $\overline{ \mathbf{r}}$ are the global position vectors obtained from the HFM and ROMs, respectively. The subscripts $\star _{X}$, $\star _{Y}$, $\star _{Z}$ indicate directional components along the $OX$, $OY$, $OZ$ axis. As can be observed in Fig. 7(a), although both the ROMs with nodal-fixed frame and mean-axis frame can reproduce the full solution with satisfactory accuracy (RMS errors below $1.5\times 10^{-4}$), the MFR-ERubin-NL is more accurate (RMS error below $2\times 10^{-5}$) than NFR-ECB-NL, for an ROM of the same size. As can be seen in Fig. 7(b), when the rotational velocity parameter $\omega _{s}$ increases from 6 to $12~\text{rad}/ \text{s}$, the REs of both NFR-ECB-NL and MFR-ERubin-NL increase since larger geometrical nonlinearities are triggered. The REs obtained from MFR-ERubin-NL are always significantly lower than their counterparts in NFR-ECB-NL for all the load cases. The mean-axis formulation (MFR-ERubin-NL) gives better results as the relative displacement is smaller than in the nodal-fixed formulation (NFR-ECB-NL). As a result, the MDs better capture nonlinear displacements in MFR-ERubin-NL.

### Model 2: 5 MW/61.5 m wind turbine blade

**Fig. 8:**
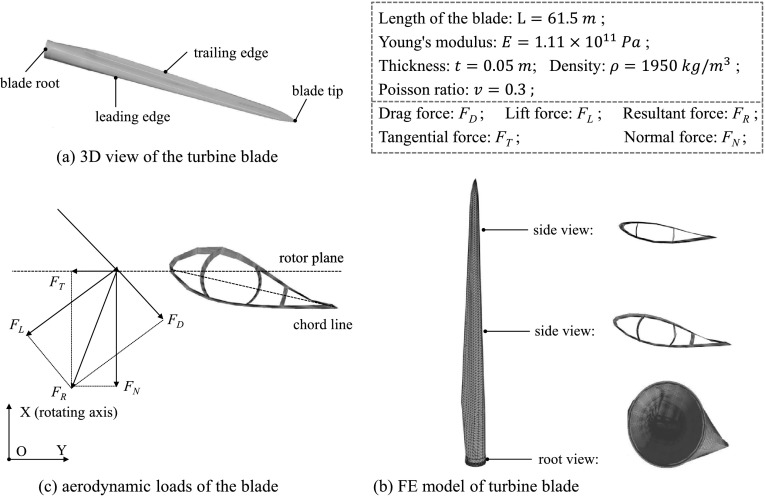
Illustration of the 61.5 m blade of the NREL 5 MW reference wind turbine. The mesh contains 3989 nodes and 8394 elements, which yields 23934 DoFs

We consider here a more complex example, namely a 61.5 m long blade of the NREL 5 MW reference wind turbine, which is originally presented in [40]. This model is constructed by assuming constant thickness and homogeneous material. The effective material properties and geometrical parameters are shown in Fig. 8. Rayleigh damping [41] is adopted: a modal damping factor of $2\%$ for the first two modes is used to determine the Rayleigh coefficients.

**Fig. 9:**
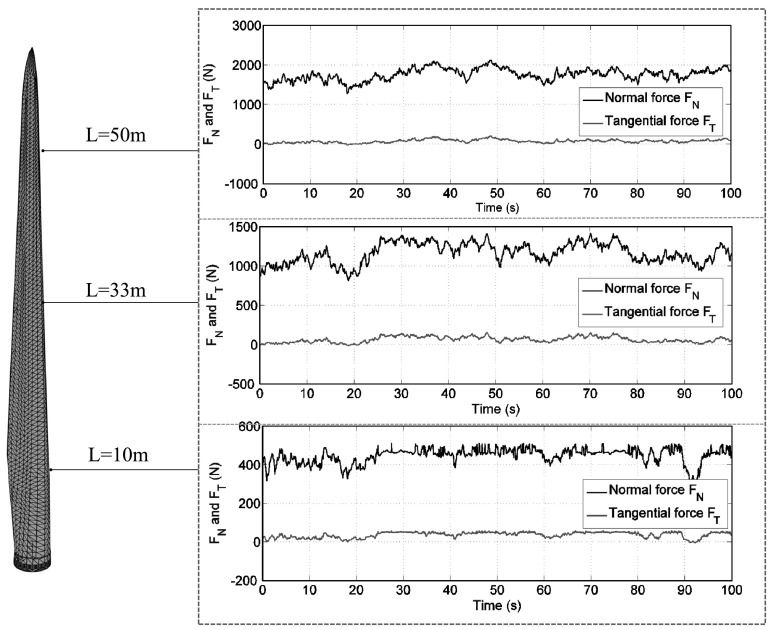
Normal forces $F_{N}$ and tangential forces $F_{T}$ at the leading edge nodes with $L=10, 33$, and 50 m for the first 100 s (Color figure online)

The aerodynamic loads experienced by the blade are calculated using the Blade Element Momentum (BEM) theory, which constitutes a broadly adopted industrial practice for design and analysis of wind turbines. The aerodynamic loads (i.e., normal force $F_{N}$ and tangential force $F_{T}$ per section) are computed as discussed in [42]. For this example, they result in prescribed, time-varying nodal forces at the leading edge of the blade. No aero-elastic interaction is here considered. For illustration, the normal force $F_{N}$ and tangential force $F_{T}$, calculated as in [42], along the leading edge nodes with length $L$ of 10, 33 and 50 m, respectively, are shown in Fig. 9.

The blade is assumed to rotate around the $x$-axis with a constant speed $\varOmega =8~\text{rad}/\text{s}$ and a physical time of 100 s is simulated. For the time integration, we use a fixed time step of 0.02 s, with updating of the tangential operator at each iteration within one time step, with a convergence criterion on the norm of the force residual relative to the norm of the internal force vector (tolerance set to $10^{-6}$).

**Fig. 10:**
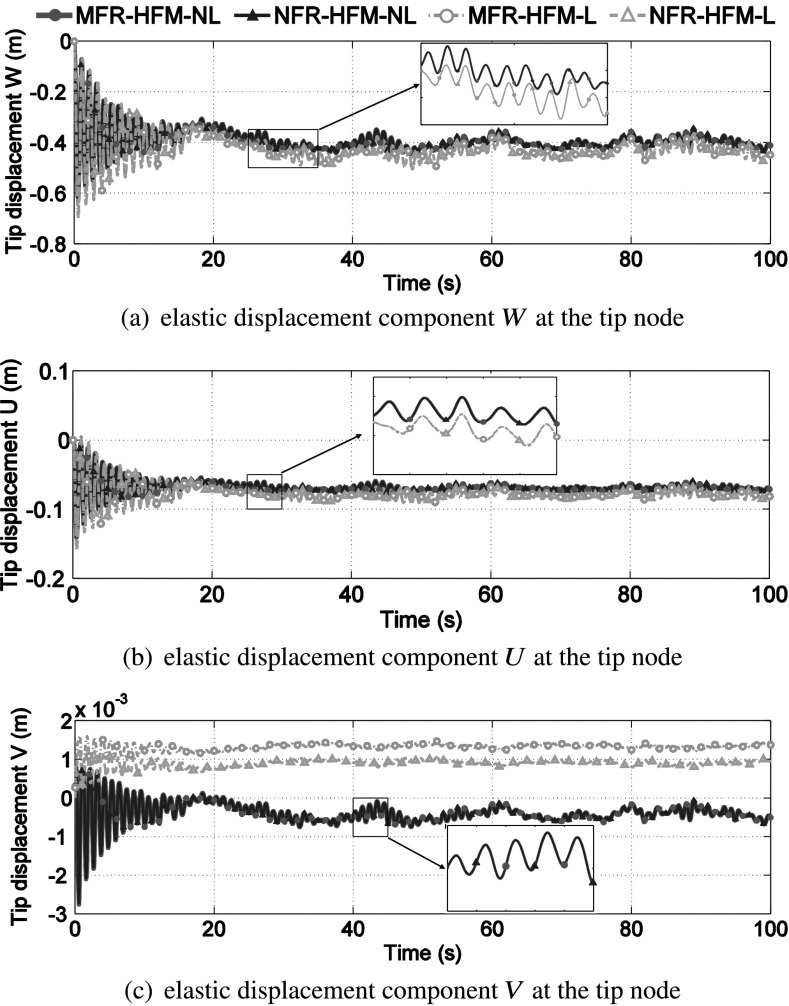
Comparison of linear and nonlinear tip displacement $W$, $U$ and $V$ of the blade tip node. A clear difference between the linear and nonlinear responses can be observed, confirming that the blade vibrates in the nonlinear regime

The tip displacement $W$, $U$ and $V$ of the blade tip node obtained from the elastically linear (NFR-HFM-L, MFR-HFM-L) and nonlinear (NFR-HFM-NL, MFR-HFM-NL) HFMs are compared in Fig. 10. The computation of the tip displacement has been shown in (51). A clear difference between the linear and nonlinear tip displacement $W$, $U$ and $V$ can be observed, confirming that the blade vibrates in the nonlinear regime. On the other hand, the relative displacements obtained from MFR-HFM-NL and NFR-HFM-NL are in good agreement.

**Fig. 11:**
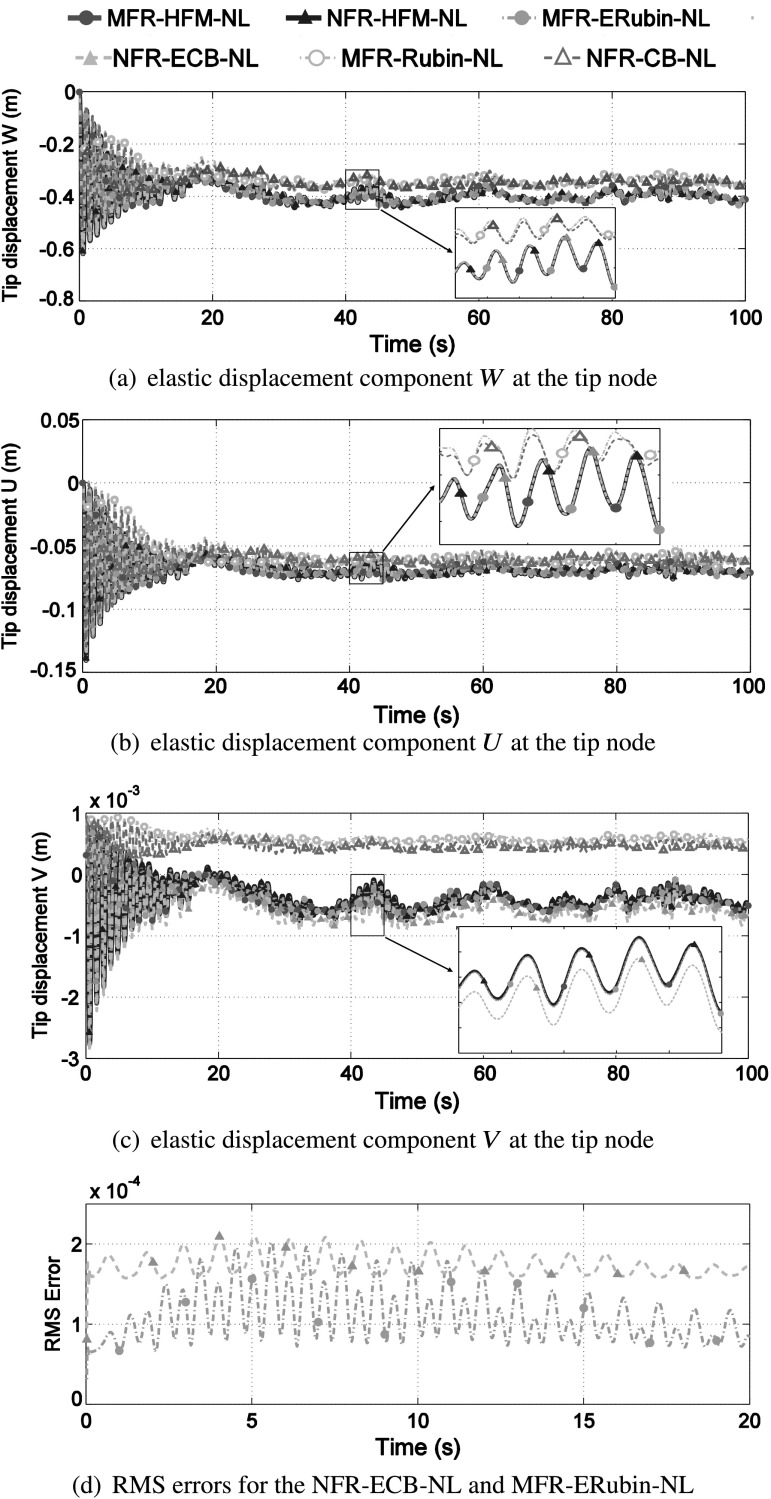
Nonlinear response of the monitored node for different ROMs. The ROMs with MDs (i.e., MFR-ERubin-NL and NFR-ECB-NL) yield much better approximations than their counterparts without MDs (i.e., MFR-Rubin-NL and NFR-CB-NL)

The different ROBs for the nonlinear analysis, for both nodal-fixed frame and mean-axis frame, are listed in Table 2. The reduced nonlinear responses obtained from different ROMs are shown in Fig. 11. As can be observed, the ROMs with MDs (i.e., MFR-ERubin-NL and NFR-ECB-NL) yield much better approximations than their counterparts without MDs (i.e., MFR-Rubin-NL and NFR-CB-NL), even though many more modes are included in these latter ones. This confirms the efficiency of using MDs to account for the nonlinear effect. Furthermore, the enhanced Rubin basis performs better than the enhanced CB counterpart in the nodal-fixed frame, even though the same number of MDs are included. The better accuracy is clearly highlighted in Fig. 11(d), where the RMS errors of the overall displacement field are compared.

**Table 2:** Number of DoFs for the 61.5 m blade model of the NREL 5 MW reference wind turbine

	Number of modes in linear ROB	Number of enriched MDs	Total DoFs
NFR-ECB-NL	10	15	25
MFR-ERubin-NL	10	15	25
NFR-CB-NL	50	0	50
MFR-Rubin-NL	50	0	50

The computational time is compared between the ROMs enriched with MDs (NFR-ECB-NL and MFR-ERubin-NL) and the HFMs (MFR-HFM-NL and NFR-HFM-NL). All simulations are performed in MATLAB^®^R2015, on a cluster equipped with 8-core Intel^®^ Xeon^®^ CPUs (E5-2630v3) @ 2.4 GHz and 128 GB RAM.

The computational cost of the HFM is denoted by $t_{\mathrm{full}}$ while the one of ROMs has been divided into three different components: (i) the construction of the reduction basis, which is regarded as *offline cost* ($t_{\mathrm{off}_{1}}$); (ii) the calculation of all the higher-order tensors as in (49), also included in the *offline cost* ($t_{\mathrm{off}_{2}}$), and (iii) the time required for time integration, which constitutes the *online cost* ($t _{\mathrm{on}}$). Clearly, the HFM does not bear any offline cost. The computational time is shown in Table 3.

**Table 3:** Computational cost for the 61.5 m wind turbine blade (100 s physical time)

Floating frame	HFM	ROM			Number of iterations	Speed up factor	
		Offline		Online			
	$t_{\mathrm{full}}$ (s)	$t_{\mathrm{off}_{1}}$ (s)	$t_{\mathrm{off}_{2}}$ (s)	$t_{\mathrm{on}}$ (s)	$\mathcal{N}$	$\mathcal{S}_{1}$	$\mathcal{S}_{2}$
Mean-axis	155307	2053	230	72	10598	2157	66
Nodal-fixed	49254	13.25	241	125	17758	394	129.87

In order to compare the time gains given by the ROMs, a speed-up factor $\mathcal{S}$ is then computed as

54 $$ \mathcal{S}=\frac{\mathcal{C}_{\mathrm{on}}t_{\mathrm{full}}}{\mathcal{C}_{\mathrm{off}}(t_{\mathrm{off} _{1}}+t_{\mathrm{off}_{2}})+\mathcal{C}_{\mathrm{on}}t_{\mathrm{on}}}, \quad \text{with } \mathcal{C}_{\mathrm{off}}+ \mathcal{C}_{\mathrm{on}}=1, $$

where $\mathcal{C}_{\mathrm{off}}$ and $\mathcal{C}_{\mathrm{on}}$ are weight factors for the offline and online stages, respectively. The offline calculation cost is neglected by setting $\mathcal{C}_{\mathrm{off}}=0$, $\mathcal{C}_{\mathrm{on}}=1$. The so obtained speed up factor, denoted as $\mathcal{S}_{1}$, is justified when the same ROM is used for many different load cases. Alternatively, one can set equal weights to offline and online costs, i.e., $\mathcal{S}_{2}: \mathcal{C}_{\mathrm{off}}=0.5$, $\mathcal{C}_{\mathrm{on}}=0.5$. This covers the limit case in which the ROM is used only once. In the context of wind turbines, a considerable number of loading conditions is prescribed by the design standard IEC 61400-1 [43], resulting in a minimum acceptable number of simulations in the order of 1880 [44]. This number grows rapidly when one or more of the parameters associated with the site environmental conditions lies outside the range of IEC reference conditions and may quickly result in up to 3,200,000 simulations [45]. The significance of online cost becomes even more pronounced in the context of digital twin technology, which constitutes the state-of-the-art approach for lifecycle management of wind turbine structures. Such technology consists in combining a virtual system model, i.e., digital twin of the wind turbine, with operational sensor data so as to afford real-time assessment of the structural condition of wind turbines, making thus $\mathcal{S}_{1}$ a decisive factor in comparison to $\mathcal{S}_{2}$.

It can be observed that $t_{\mathrm{full}}$ and $t_{\mathrm{off}_{1}}$ of the mean-axis frame are larger than their counterparts of the nodal-fixed frame, since the stiffness and mass matrices $\mathbf{M}_{mm}$ and $\mathbf{K}_{mm}$ feature a worse sparsity pattern due to the condensation of the mean axis constraints. Therefore, the eigenvalue analysis in Eq. (34), the calculation of MDs in Eq. (41), as well as tangent operator calculation in Newmark time integration are more expensive than their correspondents in the nodal fixed frame. The offline cost $t_{\mathrm{off}_{2}}$ is similar for the mean-axis frame and nodal-fixed frame ROMs, as $t_{\mathrm{off}_{2}}$ is mainly determined by the size of ROBs, i.e., the number of modes included in the reduction basis. On the contrary, $t_{\mathrm{on}}$ in the mean-axis frame is much smaller than its counterpart in the nodal-fixed frame. This is due to the fact that the MFR-ERubin-NL requires fewer iterations for a given time step because of smaller relative DoFs $\mathbf{q}_{f}$, although NFR-ECB-NL and MFR-ERubin-NL contain the same number of modes in the ROB and their corresponding computational time per iteration is similar.

## Conclusions

This paper presents a model-order reduction technique for flexible multibody systems featuring geometrically nonlinear elastic behavior. The overall motion of each body is described with the mean-axis floating frame of reference. The relative displacements of each body are then represented by a basis obtained by enhancing the standard Rubin substructuring basis with modal derivatives computed for both free vibration modes and attachment modes. This allows to accurately capture the geometrically nonlinear elastic behavior of the deformable body. When compared with a previous contribution [1], where a modal derivatives-enhanced Craig–Bampton substructuring method is applied in the nodal-fixed floating frame, the present approach offers a better representation of the nonlinearity at the interface, since the coupling between attachment modes and free-interface modes is considered.

For the reduced-order model, the modal derivatives essentially represent second-order terms of the Taylor expansion of the displacements from the undeformed configuration. As such, it is essential to minimize the relative displacements and rotations with respect to the reference frame. The mean-axis formulation indeed provides generally smaller relative displacements and rotations than their counterpart in the nodal-fixed frame, thus improving the accuracy of the reduced-order model, as shown in the numerical examples.

The method provides significant computational gains when tested on the simulation of a flexible wind turbine blade featuring about 24000 degrees of freedom. The necessary offline cost for the computation of reduction basis is higher than the one proposed obtained in [1], since the projected matrices feature worse sparsity pattern due to the embedding of the mean-axis constraints. However, the online speed-up is better for the chosen test as compared to the one achieved in [1] for a reduced-order model of equal size. This is due to the fewer iterations within a time step required for convergence, as the relative displacements with respect to the reference frame are smaller. This technique is particularly useful when several load conditions need to be simulated so that the offline cost can be amortized.

## Appendix A: The configuration-dependent mass matrix

This appendix contains a detailed derivation of the configuration depended mass matrix. By substituting (3) into (5), we can obtain

55 $$\begin{aligned} \mathcal{T}^{j} &= \frac{1}{2} \int _{V^{j}} \rho ^{j}\bigl(\dot{\mathbf{r}} ^{j}\bigr)^{\text{T}}\dot{\mathbf{r}}^{j}\,\mathrm{d}V^{j} \\ &=\frac{1}{2}\bigl(\dot{\mathbf{q}}^{j}\bigr)^{\text{T}} \int _{V^{j}} \rho ^{j} \left( \begin{bmatrix} \mathbf{I} & \boldsymbol{\mathcal{B}}^{j}\{\boldsymbol{\theta }, \mathbf{q}^{j}_{f}\} & \mathbf{A}\mathbf{N}^{j} \end{bmatrix} \right) ^{\text{T}} \left( \begin{bmatrix} \mathbf{I} & \boldsymbol{\mathcal{B}}^{j}\{\boldsymbol{\theta }, \mathbf{q}^{j}_{f}\} & \mathbf{A}\mathbf{N}^{j} \end{bmatrix} \right) \,\mathrm{d}V^{j}\dot{ \mathbf{q}}^{j} \\ &=\frac{1}{2}\bigl(\dot{\mathbf{q}}^{j}\bigr)^{\text{T}} \mathbf{M}^{j} \dot{\mathbf{q}}^{j}. \end{aligned}$$

Accordingly, the mass matrix $\mathbf{M}^{j}$ of the $j$th element can be written in a partitioned form as

56 $$\begin{aligned} \mathbf{M}^{j} &= \int _{V^{j}} \rho ^{j} \begin{bmatrix} \mathbf{I} & \boldsymbol{\mathcal{B}}^{j} & \mathbf{A}\mathbf{N}^{j}\\ & (\boldsymbol{\mathcal{B}}^{j})^{\text{T}}(\boldsymbol{\mathcal{B}}^{j}) & (\boldsymbol{\mathcal{B}}^{j})^{\text{T}}\mathbf{A}\mathbf{N}^{j}\\ \text{sym} & & (\mathbf{N}^{j})^{\text{T}}\mathbf{N}^{j} \end{bmatrix} \,\mathrm{d} V^{j} \\ &= \begin{bmatrix} \mathbf{M}^{j}_{RR} & \mathbf{M}^{j}_{R\theta }\{ \boldsymbol{\theta },\mathbf{q}_{f}^{j}\} & \mathbf{M}^{j}_{Rf}\{ \boldsymbol{\theta }\} \\ & \mathbf{M}^{j}_{\theta \theta }\{\boldsymbol{\theta },\mathbf{q} _{f}^{j}\} & \mathbf{M}^{j}_{\theta f}\{\boldsymbol{\theta }, \mathbf{q}_{f}^{j}\} \\ \text{sym} & & \mathbf{M}^{j}_{ff} \end{bmatrix} . \end{aligned}$$

It can be shown that $\mathbf{M}^{j}_{RR}$ and $\mathbf{M}^{j}_{ff}$ are constant matrices which can be calculated offline. The off-diagonal components and $\mathbf{M}^{j}_{\theta \theta }$ are configuration dependent.

With the expression of kinetic energy $\mathcal{T}^{j}$ as in (55), we can write the first two terms of the Lagrange’s equation in (7), after the FE assembly, as

57 $$ \frac{\mathrm{d}}{\mathrm{d}t} \biggl(\frac{\partial \mathcal{T}}{\partial \dot{\mathbf{q}}} \biggr)^{\text{T}}- \biggl(\frac{\partial \mathcal{T}}{ \partial \mathbf{q}} \biggr)^{\text{T}} = \mathbf{M}\ddot{\mathbf{q}}+ \dot{\mathbf{M}}\dot{\mathbf{q}}- \biggl[ \frac{\partial }{\partial \mathbf{q}} \biggl( \frac{1}{2}\dot{\mathbf{q}}^{T} \mathbf{M} \dot{\mathbf{q}} \biggr) \biggr] ^{T}. $$

We can define the quadratic velocity $\mathbf{Q}$ to be

58 $$ \mathbf{Q}=-\dot{\mathbf{M}}\dot{\mathbf{q}}+ \biggl[ \frac{\partial }{ \partial \mathbf{q}} \biggl( \frac{1}{2}\dot{\mathbf{q}}^{T} \mathbf{M} \dot{\mathbf{q}} \biggr) \biggr] ^{T}, $$

where $\mathbf{Q}$ results from the differentiation of the kinetic energy with respect to time and with respect to the body coordinates, and contains the gyroscopic and Coriolis contributions.

## Appendix B: Rigid body constraints at the interface sets

The matrix $\mathbf{L}_{v}$, as introduced in (10) to rigidize the interface DoFs, is calculated according to the relative position of each interface node with respect to the corresponding virtual node. To offer a clear view, the transformation matrix $\widehat{\mathbf{L}} _{v_{p}}$ from the DoFs $\hat{\mathbf{q}}_{b_{p}}$ of an arbitrary node in the $p$th interface set to the DoFs $\mathbf{q}_{v_{p}}$ of the corresponding virtual node has been written here

59 $$ \underbrace{ \begin{bmatrix} \hat{q}_{X,b_{p}} \\[3pt] \hat{q}_{Y,b_{p}} \\[3pt] \hat{q}_{Z,b_{p}} \\[3pt] \hat{q}_{\theta _{x},b_{p}} \\[3pt] \hat{q}_{\theta _{y},b_{p}} \\[3pt] \hat{q}_{\theta _{z},b_{p}} \end{bmatrix} } _{\hat{\mathbf{q}}_{b_{p}}} = \underbrace{ \begin{bmatrix} 1 & 0 & 0 & 0 & \Delta z & -\Delta y \\ 0 & 1 & 0 & -\Delta z & 0 & \Delta x \\ 0 & 0 & 1 & \Delta y & -\Delta x & 0 \\ 0 & 0 & 0 & 1 & 0 & 0 \\ 0 & 0 & 0 & 0 & 1 & 0 \\ 0 & 0 & 0 & 0 & 0 & 1 \end{bmatrix} }_{\widehat{\mathbf{L}}_{v_{p}}} \underbrace{ \begin{bmatrix} q_{X,v_{p}} \\ q_{Y,v_{p}} \\ q_{Z,v_{p}} \\ q_{\theta _{x},v_{p}} \\ q_{\theta _{y},v_{p}} \\ q_{\theta _{z},v_{p}} \end{bmatrix} }_{\mathbf{q}_{v_{p}}}, $$

where

$$ \Delta x = x_{b_{p}} - x_{v_{p}}, \qquad \Delta y = y_{b_{p}} - y_{v _{p}}, \qquad \Delta z = z_{b_{p}} - z_{v_{p}}, $$

and $x_{b_{p}}$, $y_{b_{p}}$, $z_{b_{p}}$ and $x_{v_{p}}$, $y_{v_{p}}$, $z _{v_{p}}$ are the nodal coordinates of the interface nodes and virtual nodes in all three directions, respectively. The vectors $\hat{\mathbf{q}}_{b_{p}}$ and $\mathbf{q}_{v_{p}}$ have been split into 3 translation component ($\star _{X}, \star _{Y}, \star _{Z}$) and 3 rotational components ($\star _{\theta _{x}}, \star _{\theta _{y}}, \star _{\theta _{z}}$), as in the subscripts. The transformation matrix $\mathbf{L}_{v}$ for the entire set of interface DoFs can then be obtained by concatenating the transformation matrices from each interface nodes in an analogous way.

## Appendix C: The constraint equations for the mean-axis frame

In this appendix, we will derive the constrained equations for the mean-axis frame with proper assumptions, as first proposed in [26]. Substituting (14) into (16), we can obtain

60 $$ \frac{\partial \mathcal{T}_{r}}{\partial \dot{\mathbf{R}}}= - \sum_{j} \int _{V^{j}}\rho ^{j} \bigl[ \dot{\mathbf{r}}^{j}- \dot{\mathbf{R}}- \dot{\mathbf{A}}\mathbf{N}^{j}\bigl( \mathbf{q}_{0}^{j}+\mathbf{q}_{f}^{j} \bigr) \bigr] \,\mathrm{d}V^{j} =\mathbf{0} $$

and

61 $$ \frac{\partial \mathcal{T}_{r}}{\partial \boldsymbol{\omega }} = - \sum_{j} \int _{V^{j}} \rho ^{j} \frac{\partial }{\partial \boldsymbol{\omega }} \bigl[ \dot{ \mathbf{A}}\mathbf{N}^{j}\bigl( \mathbf{q}_{0}^{j}+ \mathbf{q}_{f}^{j}\bigr) \bigr] ^{\text{T}} \bigl[ \dot{ \mathbf{r}}^{j}-\dot{\mathbf{R}}-\dot{\mathbf{A}}\mathbf{N}^{j} \bigl( \mathbf{q}_{0}^{j}+\mathbf{q}_{f}^{j} \bigr) \bigr] \,\mathrm{d}V^{j}=0. $$

Substituting (3) into (60) and (61), then noticing that $\mathbf{A}$ is not space dependent, we can further get that

62 $$ \frac{\partial \mathcal{T}_{r}}{\partial \dot{\mathbf{R}}}=\mathbf{0} \quad \Leftrightarrow \quad \sum _{j} \int _{V^{j}}\rho ^{j}\mathbf{N}^{j}\dot{ \mathbf{q}}_{f}^{j} \,\mathrm{d}V^{j} =\mathbf{0} $$

and

63 $$ \frac{\partial \mathcal{T}_{r}}{\partial \boldsymbol{\omega }}= \mathbf{0} \quad \Leftrightarrow \quad \sum _{j} \int _{V^{j}}\rho ^{j}\bigl(\mathbf{q}_{0}^{j}+ \mathbf{q}_{f}^{j}\bigr)^{ \text{T}}\bigl( \mathbf{N}^{j}\bigr)^{\text{T}} \frac{\partial }{\partial \boldsymbol{\omega }} \bigl( \dot{ \mathbf{A}}^{\text{T}}\mathbf{A} \bigr) \mathbf{N}^{j}\dot{ \mathbf{q}}_{f}^{j}\,\mathrm{d}V^{j} = \mathbf{0}, $$

where we can further utilize the identity [5]

64 $$ \dot{\mathbf{A}}^{\text{T}}\mathbf{A}=- \begin{bmatrix} 0 & -\omega _{3} & \omega _{2} \\ \omega _{3} & 0 & -\omega _{1} \\ -\omega _{2} & \omega _{1} & 0 \end{bmatrix} =-\widetilde{\boldsymbol{\omega }}, $$

where $\widetilde{\star }$ indicates the skew symmetric matrix of the corresponding vector. Substituting (64) into (63), we can obtain

65 $$ \sum_{j} \int _{V^{j}}\rho ^{j}\bigl(\mathbf{q}_{0}^{j}+ \mathbf{q}_{f}^{j}\bigr)^{ \text{T}}\bigl( \mathbf{N}^{j}\bigr)^{\text{T}} \frac{\partial }{\partial \boldsymbol{\omega }}\widetilde{ \boldsymbol{\omega }} \mathbf{N}^{j} \dot{\mathbf{q}}_{f}^{j} \,\mathrm{d}V^{j}=\mathbf{0} , $$

which can be further derived as

66 $$ \begin{bmatrix} (\mathbf{q}^{j}_{0}+\mathbf{q}^{j}_{f})^{\text{T}} \widetilde{\mathbf{N}}^{j}_{23}\\ (\mathbf{q}^{j}_{0}+\mathbf{q}^{j}_{f})^{\text{T}} \widetilde{\mathbf{N}}^{j}_{31}\\ (\mathbf{q}^{j}_{0}+\mathbf{q}^{j}_{f})^{\text{T}} \widetilde{\mathbf{N}}^{j}_{12} \end{bmatrix} \dot{ \mathbf{q}}_{f} =\mathbf{0}, \quad\text{with } \widetilde{ \mathbf{N}}^{j}_{kl}= \int _{V^{j}}\rho ^{j} \bigl[ \bigl( \mathbf{N}^{j}_{k}\bigr)^{\text{T}}\mathbf{N}^{j}_{l}- \bigl(\mathbf{N}^{j}_{l}\bigr)^{ \text{T}} \mathbf{N}^{j}_{k} \bigr] \,\mathrm{d} V^{j}, $$

where $\mathbf{N}^{j}_{k}$ and $\mathbf{N}^{j}_{l}$ are the $k$th and $l$th rows of the shape function $\mathbf{N}^{j}$. In order to linearize the mean-axis condition, the simplification in the reference [26] aims to neglecting the higher order terms in (63) by stating

67 $$ \begin{bmatrix} (\mathbf{q}^{j}_{f})^{\text{T}}\widetilde{\mathbf{N}}^{j}_{23}\\ (\mathbf{q}^{j}_{f})^{\text{T}}\widetilde{\mathbf{N}}^{j}_{31}\\ (\mathbf{q}^{j}_{f})^{\text{T}}\widetilde{\mathbf{N}}^{j}_{12} \end{bmatrix} \dot{ \mathbf{q}}_{f} =\mathbf{0}. $$

Notice that $\widetilde{\mathbf{N}}^{j}_{kl}$ is a skew symmetric matrix. Therefore, assumption (67) can be justified on the basis that velocity $\dot{\mathbf{q}}_{f}^{j}$ occur approximately collinear with displacement $\mathbf{q}^{j}_{f}$, and hence the cross product vanishes.

With this assumption, the mean-axis condition can be linearized as

68 $$ \frac{\partial \mathcal{T}_{r}}{\partial \dot{\mathbf{R}}}=\mathbf{0} \quad \Rightarrow \quad \mathbf{S}^{j}_{1} \dot{\mathbf{q}}_{f}^{j} =\mathbf{0} \quad \text{with } \mathbf{S}^{j}_{1}=\sum _{j} \int _{V^{j}}\rho ^{j} \mathbf{N}^{j} \,\mathrm{d}V^{j} $$

and

69 $$ \frac{\partial \mathcal{T}_{r}}{\partial \boldsymbol{\omega }}= \mathbf{0} \quad \Rightarrow \quad \mathbf{S}_{2} \dot{\mathbf{q}}_{f} =\mathbf{0}, \quad \text{with } \mathbf{S}^{j}_{2}= \begin{bmatrix} (\mathbf{q}^{j}_{0})^{\text{T}}\widetilde{\mathbf{N}}^{j}_{23}\\ (\mathbf{q}^{j}_{0})^{\text{T}}\widetilde{\mathbf{N}}^{j}_{31}\\ (\mathbf{q}^{j}_{0})^{\text{T}}\widetilde{\mathbf{N}}^{j}_{12} \end{bmatrix} . $$

Equations (68) and (69) can be further expressed, with a classic FE assembly, as

70 $$ \begin{bmatrix} \mathbf{S}_{1}\\ \mathbf{S}_{2} \end{bmatrix} \dot{\mathbf{q}}_{f} =\mathbf{0} \quad \Leftrightarrow \quad \mathbf{S}\dot{ \mathbf{q}}_{f}=\mathbf{0}, $$

where $\mathbf{S}_{1} \in \mathbb{R}^{3\times n}$ and $\mathbf{S}_{2} \in \mathbb{R}^{3 \times n}$ are the precomputed inertia integrals, which can be assembled over the elementary component $\mathbf{S}_{1} ^{j}\in \mathbb{R}^{3\times n_{e}}$ and $\mathbf{S}_{2}^{j} \in \mathbb{R}^{3 \times n_{e}}$. The mean-axis condition in (70) implies that the mean-axis condition can be written in terms of only $\dot{\mathbf{q}}_{f}$ that have been defined locally w.r.t. the body axis.

## Footnotes

1 Note that the number of obtainable MDs grows quadratically with respect to the number of modes contained in the Rubin basis $\mathbf{X}$. In [30], two MDs selection criteria are discussed with the goal of selecting only a relevant subset of all possible MDs and therefore reduce the ROB size.
